# Activation of Piezo1 sensitizes cells to TRAIL-mediated apoptosis through mitochondrial outer membrane permeability

**DOI:** 10.1038/s41419-019-2063-6

**Published:** 2019-11-04

**Authors:** Jacob M. Hope, Maria Lopez-Cavestany, Wenjun Wang, Cynthia A. Reinhart-King, Michael R. King

**Affiliations:** Department of Biomedical Engineering, Vanderbilt University, 5824 Stevenson Center, Nashville, TN 37235 USA

**Keywords:** Targeted therapies, Apoptosis

## Abstract

TRAIL specifically induces apoptosis in cancer cells without affecting healthy cells. However, TRAIL’s cancer cytotoxicity was insufficient in clinical trials. Circulatory-shear stress is known to sensitize cancer cells to TRAIL. In this study, we examine the mechanism of this TRAIL sensitization with the goal of translating it to static conditions. GsMTx-4, a Piezo1 inhibitor, was found to reduce shear stress-related TRAIL sensitization, implicating Piezo1 activation as a potential TRAIL-sensitizer. The Piezo1 agonist Yoda1 recreated shear stress-induced TRAIL sensitization under static conditions. A significant increase in apoptosis occurred when PC3, COLO 205, or MDA-MB-231 cells were treated with Yoda1 and TRAIL in combination, but not in Bax-deficient DU145 cells. Calpastatin inhibited apoptosis in Yoda1-TRAIL treated cells, indicating that calpain activation is necessary for apoptosis by Yoda1 and TRAIL. Yoda1 and TRAIL treated PC3 cells showed increased mitochondrial outer membrane permeability (MOMP), mitochondrial depolarization, and activated Bax. This implies that Piezo1 activation sensitizes cancer cells to TRAIL through a calcium influx that activates calpains. The Calpains then induce MOMP by enhancing Bax activation. From these experiments a computational model was developed to simulate apoptosis for cells treated with TRAIL and increased calcium. The computational model elucidated the proapoptotic or antiapoptotic roles of Bax, Bcl-2, XIAP, and other proteins important in the mitochondrial-apoptotic signaling pathway.

## Introduction

With 8 million cancer-related deaths per year, major breakthroughs in cancer therapy are needed^1^. Tumor-necrosis-factor-α (TNF-α)-related apoptosis-inducing ligand (TRAIL) is a promising cancer therapy discovered by Wiley et al. in 1995^2^. TRAIL induces apoptosis specifically in cancer cells, while sparing healthy cells thus minimizing side effects^3^. This prompted multiple clinical trials using TRAIL^4–7^. The clinical trials showed that TRAIL lacked the necessary cytotoxicity for clinical relevance. Thus, focus has shifted to discovering compounds that enhance TRAIL’s cytotoxicity while maintaining its specificity^8^.

TRAIL induces apoptosis in cancer cells by binding to death receptors 4 and 5 (DR4/5)^3^. Cancer cells will undergo different forms of TRAIL-mediated apoptosis dependent on whether they are type I or II cells^9^. Type I cells follow the extrinsic pathway. When TRAIL binds to DR4/5, the death-induced signaling complex (DISC) is formed, activating caspase 8. Caspase 8 activates caspase 3, which cleaves functional proteins needed for cell survival^10^. In type II cells the extrinsic pathway cannot commit a cell to apoptosis. Caspase 8 additionally cleaves Bid to truncated Bid (tBid) leading to activation of the intrinsic pathway^11^. TBid activates this pathway by inhibiting Bcl-2 and activating Bax to form pores in the mitochondria. These pores lead to mitochondrial outer membrane permeability (MOMP) and the release of the apoptogenic proteins cytochrome c and Smac^12–15^.

Previously cancer cells have been sensitized to TRAIL-mediated apoptosis when exposed to circulatory levels of shear stress^16^. One potential explanation for this shear stress mechanism is the activation of mechanosensitive ion channels (MSCs), specifically the MSC Piezo1. Piezo1 is an MSC that opens in response to mechanical stimuli, such as shear stress and like other MSCs has been previously associated with proapoptotic effects^17–21^. Furthermore, Piezo1 has a small molecule agonist known as Yoda1, meaning Piezo1’s activity can be translated to static conditons^22^.

The proapoptotic effects of Piezo1 and other MSCs have primarily been associated with calcium influx^19,20^. One pathway by which calcium induces apoptosis is by causing mitochondrial dysfunction. Calcium influx can cause mitochondrial dysfunction by activating calpains, proteolytic enzymes that cleave Bcl-2 and process Bid to tBid, inducing intrinsic apoptosis^23–25^.

The mechanism through which shear stress sensitizes cancer cells to TRAIL-mediated apoptosis has not yet been elucidated, nor has a method of exploiting shear stress TRAIL sensitization within tumors been identified. In this study, we demonstrate the role of Piezo1 in shear stress-induced TRAIL sensitization of cancer cells, translate Piezo1’s TRAIL-sensitizing role to static conditions using Yoda1, and explore the mechanism of Piezo1 and TRAIL’s apoptotic synergy using Yoda1 experiments and a new computational model.

## Results

### Shear sensitization of PC3 cells to TRAIL-mediated apoptosis is reduced by MSC inhibition

**Fig. 1:**
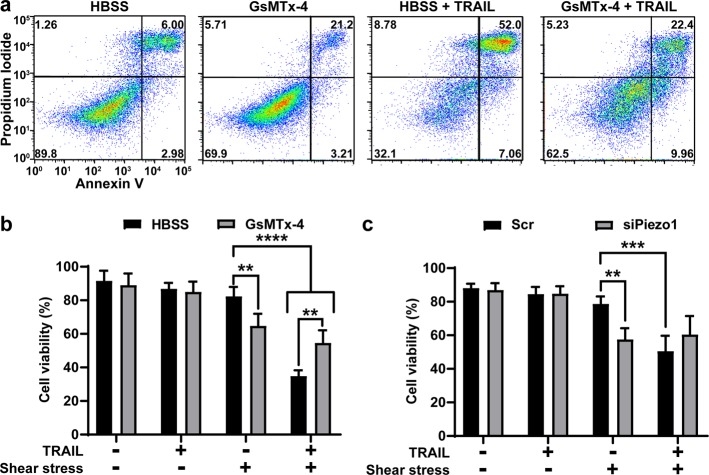
Shear stress sensitization of PC3 cells to TRAIL-mediated apoptosis. **a** Annexin-V flow plots of PC3 cells treated with shear stress and combinations of HBSS or 10 µM GsMTx-4 and 250 ng/mL TRAIL. **b** Cell viabilities for PC3 cells treated with shear stress, HBSS, GsMTx-4, or TRAIL (*n* = 4). **c** Cell viabilities of PC3 cells with Piezo1 or scrambled siRNA after treatment with shear stress and TRAIL (*n* = 4). **a** One representative experiment of four independent experiments. **b**, **c** Means and SD of four independent experiments. Statistical significance determined by one-tailed ANOVA. **p < 0.01, ***p < 0.005, ****p < 0.001

Cell viability was measured after PC3 (prostate) cells were treated with 250 ng/mL TRAIL, shear stress of 2.0 dyn/cm^2^, and 10 µM GsMTx-4 for 4 h (Fig. 1a). The percent of viable cells was determined using Annexin-V/propidium iodide (PI) staining. Cells negative for Annexin-V and PI were considered viable. PC3 cells treated with 250 ng/mL TRAIL under static conditions showed a negligible drop in cell viability. When the cells were exposed to shear stress of 2.0 dyn/cm^2^ and TRAIL, a significant decrease in cell viability to 34.8% was found (Fig. 1b). Shear stress exposure alone did not cause a major shift in viability. The pharmacological inhibitor of MSCs, GsMTx-4, significantly increased viability by 19.8% when used with shear stress and TRAIL. GsMTx-4 treated cells exhibited a reduced viability of 64.8% when exposed to shear stress (Fig. 1b). This indicates that some of the apoptosis detectable in the shear stress-GsMTx-4-TRAIL treated group is not due to TRAIL. To account for this possibility, shear stress-induced TRAIL sensitization was calculated for the GsMTx-4 and non-GsMTx-4 shear stress-TRAIL treated cells (Supplementary Fig. 1a). Shear stress-induced TRAIL sensitization was calculated by subtracting the cell viability of the TRAIL treated group from its non-TRAIL-treated counterpart and then dividing by the viability of the non-TRAIL-treated group. Cells exposed to only shear stress showed a TRAIL sensitization of 57.7%, whereas cells experiencing GsMTx-4 and shear stress had 13.4% (Supplementary Fig. 1a). These results suggest that MSCs play a role in shear stress sensitization of cancer cells to TRAIL.

To determine if Piezo1 specifically plays a role in this shear stress sensitization, Piezo1 expression was confirmed in PC3 cells via flow cytometry (Supplementary Fig. 2). Piezo1 was knocked down using siRNA, with knockdown confirmed using western blot (Supplementary Fig. 3a). No changes in TRAIL sensitivity occurred for siPiezo1 or scrambled PC3 cells under static conditions. The scrambled control was consistent with shear stress increasing TRAIL-mediated apoptosis with a cell viability of 50.6% (Fig. 1c). There was no significant increase in viability between the siPiezo1 cells treated with TRAIL and shear stress to the scrambled cells with TRAIL and shear stress (Fig. 1c). SiPiezo1 cells treated with shear stress showed a lower cell viability comparable to the siPiezo1 cells treated with TRAIL and shear stress (Fig. 1c). This suggests that the reduced cell viability of the siPiezo1 PC3 cells, when treated with shear stress and with TRAIL, is due to shear stress. When calculating TRAIL sensitization, the sensitization was 35.8% and −5.1% for the scrambled cells and the siPiezo1 cells, respectively (Supplementary Fig. 1b).

### Piezo1 activation by Yoda1 enhances TRAIL-mediated apoptosis

**Fig. 2:**
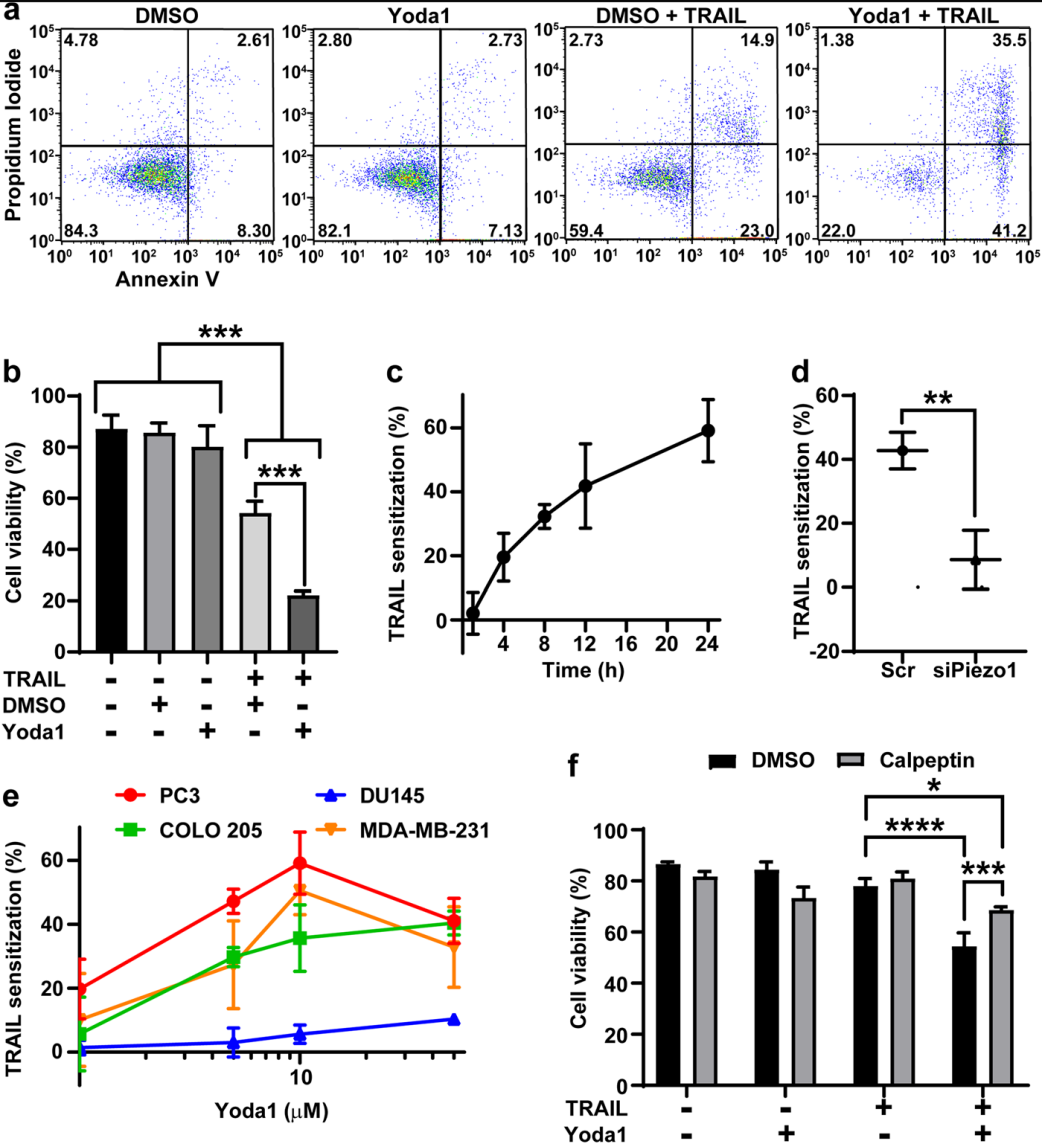
Yoda1 sensitizes cancer cells to TRAIL-mediated apoptosis. **a** Representative flow plots of Annexin-V assays of PC3 cells after treatments with combinations of 0.1% DMSO or 10 µM Yoda1 and 50 ng/mL TRAIL treatments. **b** Average cell viabilities of PC3 cells treated with DMSO or Yoda1 and TRAIL (*n* = 3). **c** TRAIL sensitization of PC3 cells by Yoda1 at 1, 4, 8, 12, and 24 h timepoints (*n* = 3). **d** TRAIL sensitization of PC3 cells by Yoda1 after siRNA knockdown of Piezo1 (*n* = 3). **e** TRAIL sensitization of PC3, DU145 (100 ng/mL), COLO 205 (10 ng/mL), and MDA-MB-231 (50 ng/mL) cells treated with 1, 5, 10, and 50 µM Yoda1 (*n* = 3). **f** PC3 cells treated with Yoda1 and TRAIL and the addition of calpeptin (*n* = 3). **a** One representative experiment of three independent experiments. **b**–**f** Means and SD of three independent experiments. Statistical analysis performed using one-tailed ANOVA (**b**, **f**) and two-tailed unpaired *t*-test (**d**). *p < 0.05, **p < 0.01, ***p < 0.005, ****p < 0.001

Piezo1 activation by Yoda1 in PC3 cells was confirmed using flow cytometry to track intracellular calcium by ratiometric fluorescence of Fluo-4 and Fura Red (Supplementary Fig. 3). PC3 cells were treated with 10 µM Yoda1 or DMSO and 50 ng/mL TRAIL (Fig. 2a). Neither Yoda1 nor DMSO caused a significant increase in apoptosis (Fig. 2b). The TRAIL and DMSO treatment group had significantly increased apoptosis with a viability of 54.3%. The Yoda1-TRAIL group had a viability of 22.2% (Fig. 2b). To assess the rate of TRAIL sensitization, PC3 cells were treated with Yoda1 or DMSO and TRAIL for 1, 4, 8, 12, or 24 h. TRAIL sensitization by Yoda1 was calculated by subtracting the cell viability of Yoda1-TRAIL treated cells from that of DMSO-TRAIL treated cells and dividing by the viability of DMSO-TRAIL treated cells. Sensitization was evident by 4 h and continued to increase over 24 h (Fig. 2c). To confirm if Yoda1 sensitizes cancer cells through Piezo1 activation, Piezo1 was inhibited using siRNA knockdown. TRAIL sensitization of PC3 cells treated with scrambled siRNA was 42.7%, whereas the siPiezo1 treated cells showed a sensitization of 8.6% (Fig. 2d).

Piezo1 expression was confirmed in COLO 205, DU145, and MDA-MB-231 cancer cell lines to determine if Yoda1-TRAIL sensitization occurs in other cancer cell lines (Supplementary Fig. 2). Yoda1-TRAIL sensitization was measured for PC3, COLO 205, DU145, and MDA-MB-231 cells for 1–50 µM Yoda1 (Fig. 2e). PC3, COLO 205, and MDA-MB-231 cells showed significant TRAIL sensitization of 59.2, 40.4, and 50.6%, respectively. Significant sensitization for these cell lines began at 5 µM Yoda1. Bax-deficient DU145 cells had a lower level of TRAIL sensitization, only reaching a value of 10.4% at 50 µM Yoda1 (Fig. 2d)^26^. Yoda1 and TRAIL were also tested against HUVEC cells as a non-cancerous control. HUVECs were sensitized to TRAIL-mediated apoptosis by Yoda1 (Supplementary Fig. 5).

Microarray Piezo1 expression of the four cancer cell lines was gathered from the CBioportal to correlate its effects on Yoda1-TRAIL sensitization^27,28^. Piezo1 expression and TRAIL sensitization had a Spearman’s correlation coefficient of −0.4, indicating Yoda1-induced TRAIL sensitization does not correlate with the amount of Piezo1 present (Supplementary Fig. 6a). The siRNA knockdown results indicate a certain level of expression is necessary, however (Fig. 2d). Yoda1-TRAIL sensitization had a Spearman’s correlation coefficient of 0.8 with Bcl-2 expression (Supplementary Fig. 6b). This suggests that Piezo1 activation acts through the intrinsic pathway to enhance TRAIL-mediated apoptosis. Calpains induce apoptosis by regulating Bcl-2 and are activated by calcium^23^. PC3 cells were treated with Yoda1, TRAIL, and 1 µM calpeptin, a calpain inhibitor for 12 h. Cell viability was significantly increased for cells treated with TRAIL, Yoda1, and calpeptin compared to TRAIL-Yoda1 treated cells (Fig. 2e).

### Yoda1 and TRAIL destabilize the mitochondria

**Fig. 3:**
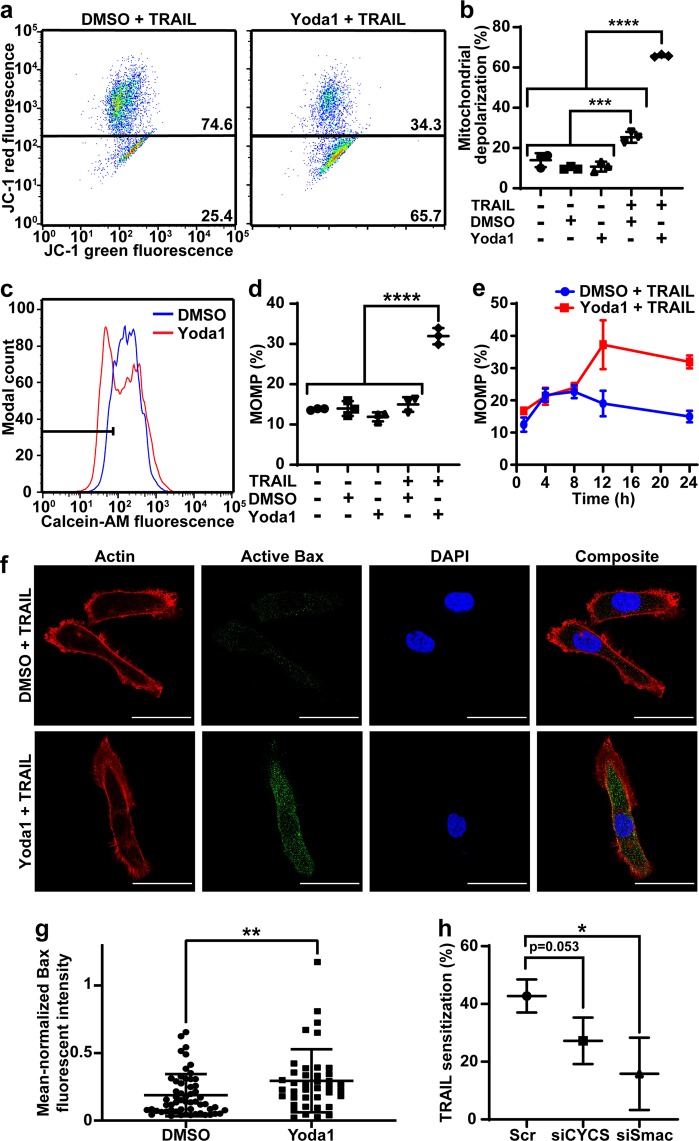
Yoda1 and TRAIL induce mitochondrial dysfunction. **a** Representative flow plots of JC-1 assay after Yoda1 or DMSO and TRAIL treatment. **b** Percent of cells with depolarized mitochondria after DMSO or Yoda1 and TRAIL treatment (*n* = 3). **c** Flow plots of MOMP due to DMSO or Yoda1 and TRAIL treatment. **d** Average MOMP of PC3 cells after treatment with DMSO or Yoda1 and TRAIL (*n* = 3). **e** MOMP of PC3 cells treated with DMSO or Yoda1 and TRAIL at 1, 4, 8, 12, and 24 h timepoints (*n* = 3). **f** Representative images of Bax activation of PC3 cells treated with DMSO or Yoda1 and TRAIL. The red channel is actin, green is active Bax, and blue is DAPI. Scale bars = 20 µm. **g** Fluorescent intensity of active Bax in PC3 cells treated with DMSO and TRAIL (*n* = 57) or Yoda1 and TRAIL (*n* = 40). **h** TRAIL sensitization of PC3 cells when treated with Yoda1 after scrambled siRNA, cytochrome c (CYCS) and Smac knockdown. **a**, **c**, **f** One representative experiment of three independent experiments. **b**, **d**, **e**, **g**, **h** Means and SD of three independent experiments. Statistical analysis was done using one-tailed ANOVA (**b**, **d**) and two-tailed unpaired *t*-test (**g**, **h**). *p < 0.05, ***p < 0.005, ****p < 0.001

Mitochondrial depolarization and MOMP was measured in PC3 cells to determine if Yoda1-TRAIL sensitization is due to the intrinsic pathway^29^. Mitochondrial depolarization was detected as a decrease in JC-1 red fluorescence. The DMSO-TRAIL group showed a significant but minimal increase in depolarization compared to the control cells with depolarization of 25.4%. Yoda1-TRAIL treated cells showed a significant mitochondrial depolarization of 65.7% (Fig. 3a, b). MOMP was measured using the calcein-CoCl_2_ assay where reduced calcein fluorescence indicates MOMP (Fig. 3c). DMSO-TRAIL treated cells had a similar level of MOMP to the other controls of 15.0%. Yoda1-TRAIL treated cells had MOMP occurrence of 31.9% (Fig. 3d). MOMP was measured at various timepoints of 1, 4, 8, 12, and 24 h for treated PC3 cells. Yoda1-TRAIL treated cells had the same value of MOMP as DMSO-TRAIL treated cells until 12 h, where a significant increase in MOMP occurred (Fig. 3e).

MOMP is caused by either mitochondrial permeability transition pore (mPTP) opening or Bax activation^13^. To determine the mechanism of MOMP, PC3 cells were treated with mPTP inhibitors, cyclosporin a (CsA) and bongkrekic acid (BKA), or the Bax channel inhibitor, Bax channel blocker (BCB). CsA and BCB enhanced TRAIL-sensitization by Yoda1 and BKA had no effect (Supplementary Fig. 7). Active Bax was measured using an antibody designed against the active conformation to determine if Yoda1 and TRAIL enhance Bax activation (Fig. 3f). Yoda1-TRAIL treated PC3 cells had significantly increased active Bax-fluorescence intensity compared to DMSO-TRAIL treated cells, suggesting that Yoda1 and TRAIL induce MOMP by Bax activation (Fig. 3g).

Cytochrome c (CYCS) and Smac were inhibited by siRNA to determine if MOMP was necessary for Yoda1-TRAIL sensitization. Knockdown of these proteins reduced TRAIL sensitization from 42.7% for the scrambled-siRNA treated cells to 27.2% and 15.8% for siCYCS and siSmac, respectively (Fig. 3h). The reduction of TRAIL sensitization of siCYCS treated cells was not statistically significant. Knockdown was confirmed via western blot (Fig. S3b, c).

### Computational model: Yoda1 and TRAIL act synergistically to induce MOMP

**Fig. 4:**
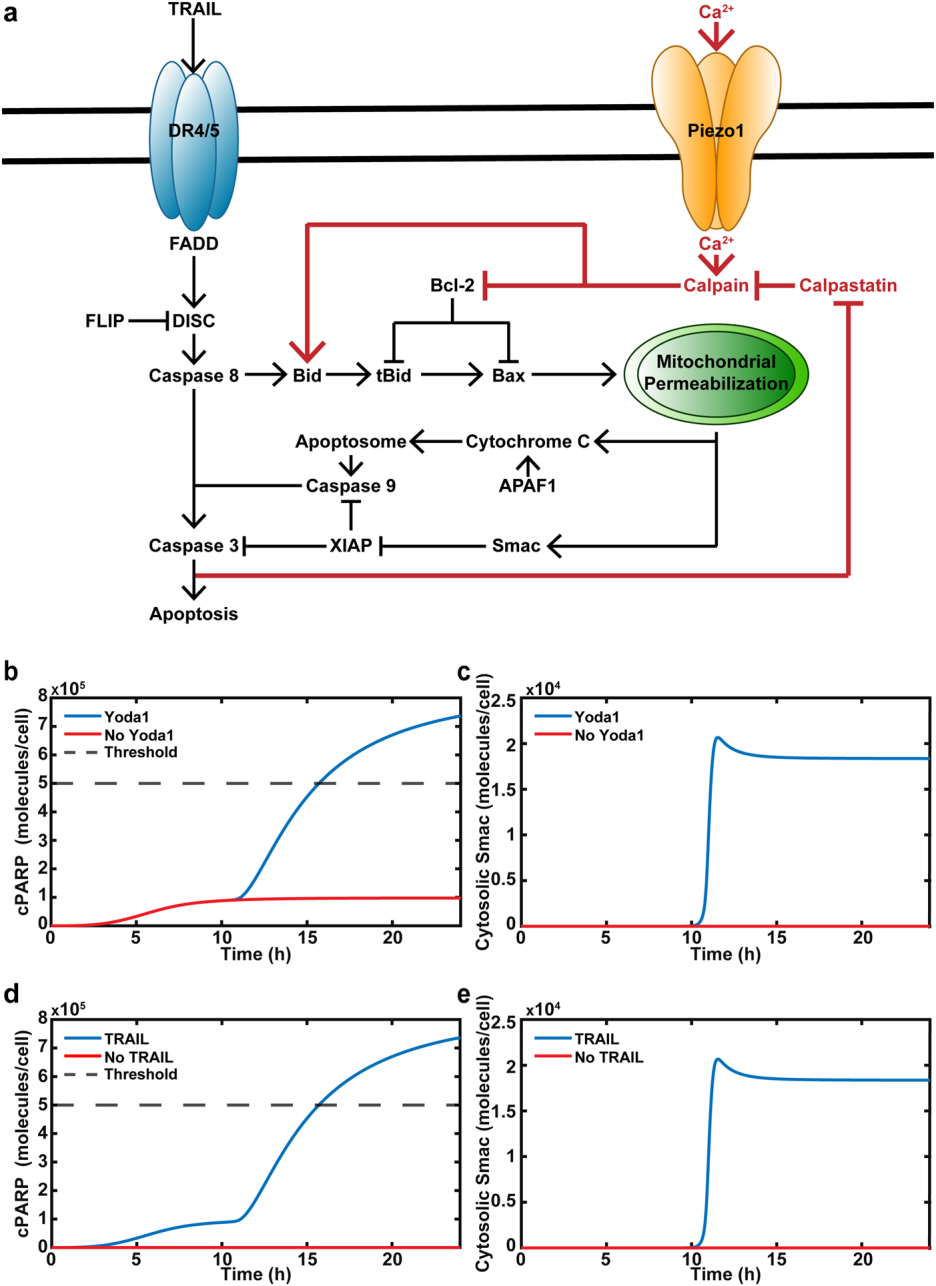
Baseline computational model of Yoda1 and TRAIL synergy. **a** Schematic of calcium and TRAIL-mediated apoptosis. Dark red coloring indicates additions to the computational model. **b** Apoptosis or cPARP concentration of cancer cells treated with TRAIL with or without Yoda1. Dashed line represents the threshold of cPARP at which cancer cells are considered apoptotic. **c** Time of MOMP determined by release of Smac, which follows MOMP for cancer cells treated with TRAIL with or without Yoda1. **d** Apoptosis of cancer cells treated with Yoda1 with or without TRAIL. **e** Time of MOMP of cancer cells treated with Yoda1 with or without TRAIL

Figure 4a depicts the mechanism of how Yoda1 and TRAIL increase apoptosis. It was determined that Yoda1 sensitizes cancer cells to TRAIL via calpains by cleaving Bcl-2 and truncating Bid. This leads to Bax activation, causing MOMP. Cleaved PARP (cPARP) is indicative of apoptosis and is used to indicate if a cancer cell underwent apoptosis in 24 h in the computational model^30^. The threshold for a cell to be considered apoptotic was if cPARP concentration reached 5*10^5^ molecules per cell. MOMP was modeled using the concentration of cytosolic Smac.

The computational model was used to determine how Yoda1 and TRAIL act synergistically to induce apoptosis. When TRAIL was used as a monotherapy, cPARP was generated, but the concentration did not reach a critical level (Fig. 4b). When Yoda1 was included, the apoptotic response initially mirrored TRAIL when used as a monotherapy, but at 12 h cPARP was amplified (Fig. 4b). Before the cPARP concentration began increasing in the Yoda1-TRAIL simulation, MOMP occurred with Smac release beginning at 10 h and reaching its maximum at 12 h. (Fig. 4b, c). The simulation shows that TRAIL-Yoda1 synergy is due to the mitochondrial dysfunction. The simulation was also performed without TRAIL (Fig. 4d, e). In the absence of TRAIL, but with Yoda1 present, there was no increase in cPARP concentration (Fig. 4d). MOMP was lost when no TRAIL was present (Fig. 4e).

### Computational model: cooperativity of TRAIL and calcium

**Fig. 5:**
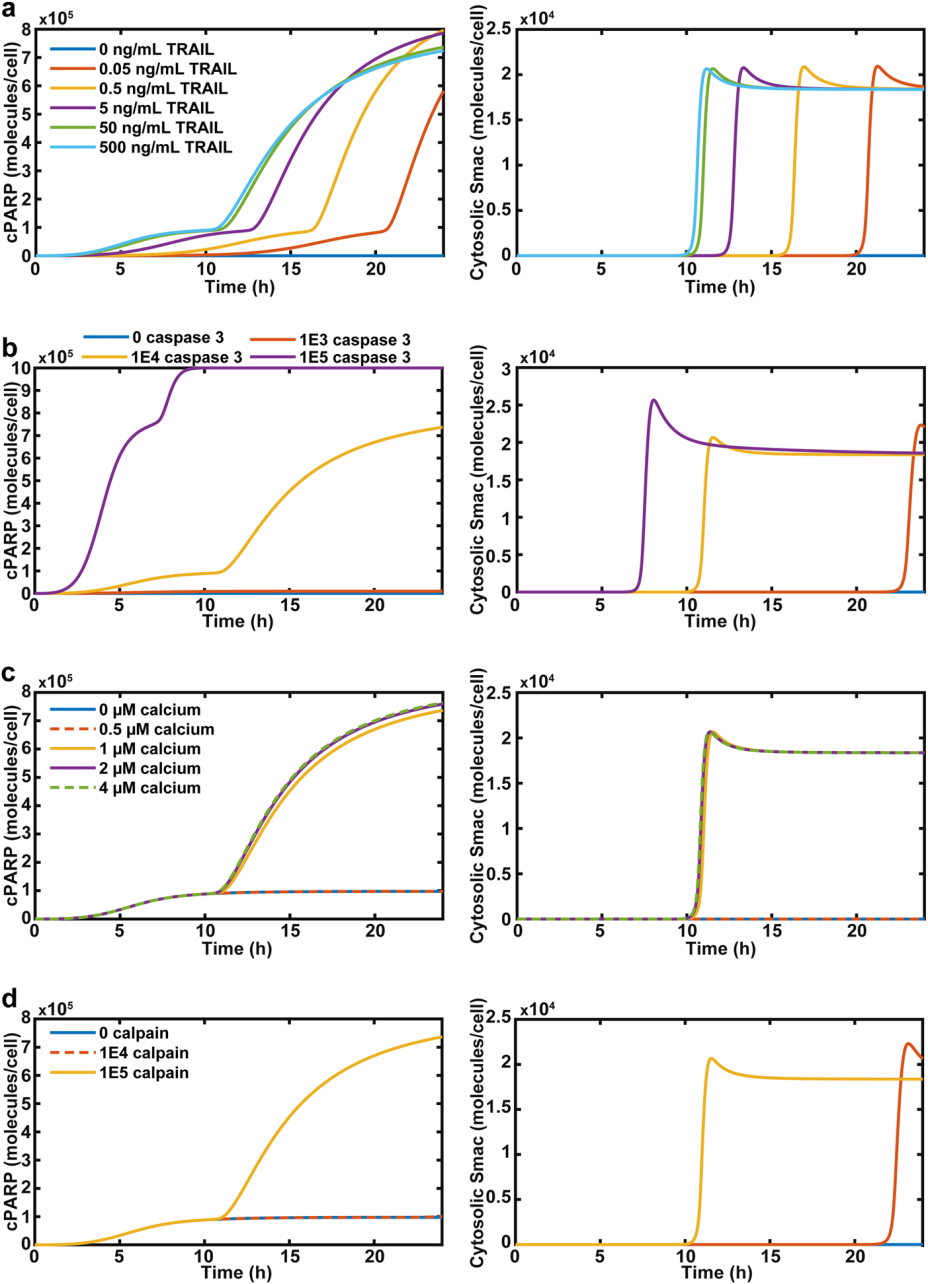
Cooperativity of TRAIL, caspase 3, calcium, and calpain on apoptosis. **a** Apoptosis and MOMP simulation of cancer cells treated with various concentrations of TRAIL and constant calcium. **b** Simulation of apoptosis and MOMP of cancer cells treated with constant calcium and TRAIL, but altered expressions of caspase 3. **c** Apoptosis and MOMP of cancer cells treated with constant TRAIL concentrations, but variable calcium concentrations. **d** Apoptosis and MOMP of cancer cells treated with constant calcium and TRAIL with differential expression of calpain

The TRAIL initial condition was varied from 0 ng/mL to 500 ng/mL. For TRAIL concentrations greater than 0, cells underwent apoptosis in 24 h, but the time of apoptosis varied (Fig. 5a). The 0.05 and 0.5 ng/mL TRAIL treatments do not produce a significant rise in cPARP until 21 and 17 h, respectively (Fig. 5a). The 50 and 500 ng/mL TRAIL cells exhibited a rise in cPARP at 12 h. The increase in cPARP for each cell occurs post-MOMP. The lower the TRAIL concentration, the later MOMP occurs, demonstrating the need for MOMP in TRAIL-mediated apoptosis (Fig. 5a).

Caspase 3 is downstream of TRAIL activating the extrinsic pathway and enhances calpain activity by degrading calpastatin, and this was included in the model (Fig. 4a)^31^. When caspase 3 expression was reduced, cPARP was lowered to a negligible level because there was not enough active caspase 3 to cleave PARP and induce MOMP (Fig. 5b). When caspase 3 was over-expressed, MOMP occurred at an earlier time and amplified cPARP. However, cells reached the threshold for apoptosis before MOMP and the amplification occurred (Fig. 5b).

Calcium concentration was varied as a proxy for different concentrations of Yoda1 treatment. Increasing calcium concentration past 1 µM had essentially no effect on enhancing TRAIL-mediated apoptosis. There was a negligible increase in cPARP and MOMP occurred only slightly sooner (Fig. 5c). Calpain expression was also varied to determine its role in this apoptotic process. Decreased calpain expression increased the time required for a cancer cell to undergo MOMP and apoptosis, and removed the effect of calcium sensitization of cancer cells to TRAIL (Fig. 5d).

### Computational model: role of intrinsic pathway proteins

**Fig. 6:**
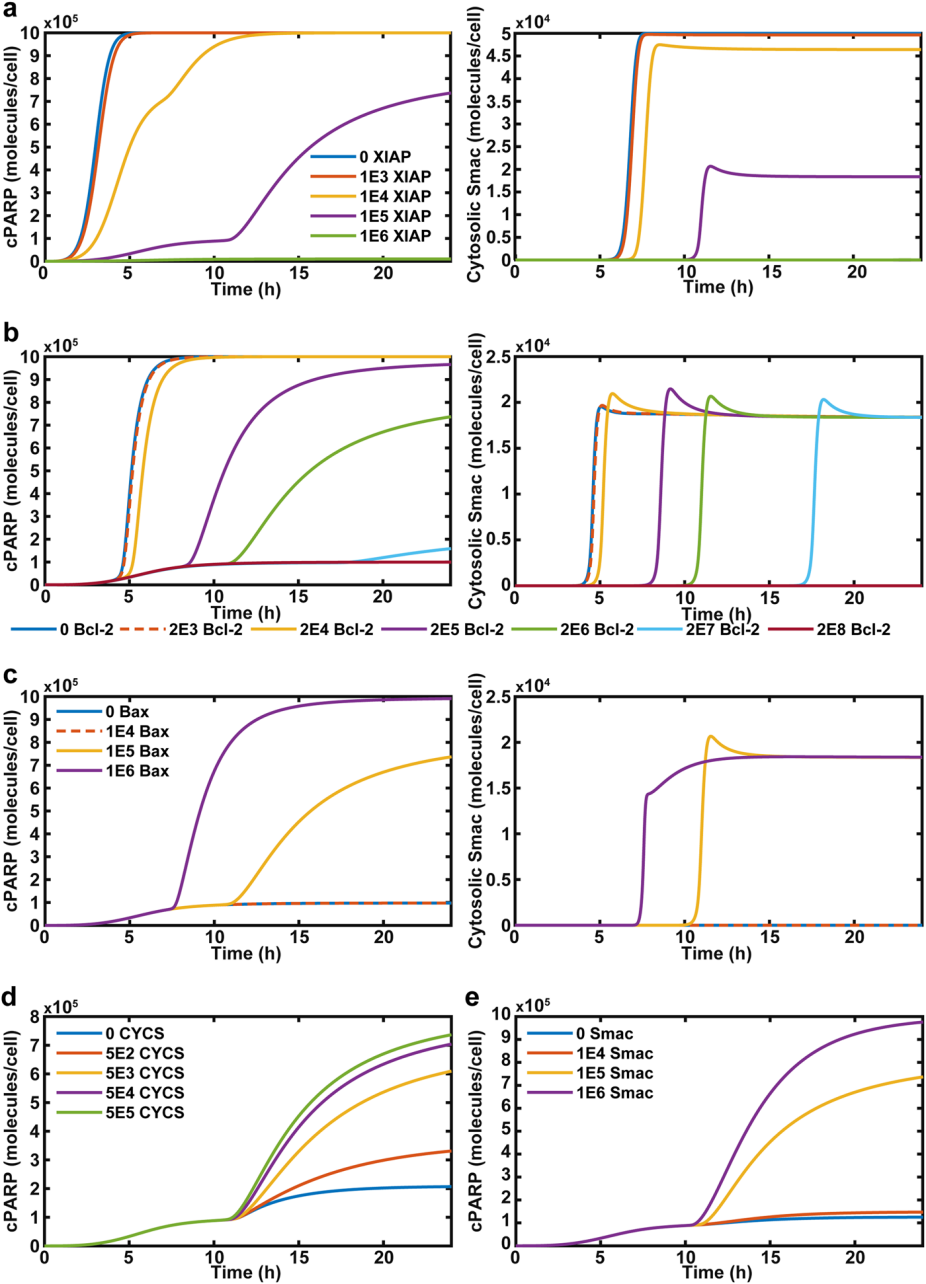
Simulation of altered initial conditions of intrinsic-apoptotic pathway proteins. **a** Simulation of apoptosis and MOMP of cancer cells with differential expressions of XIAP. **b** Apoptosis and MOMP of cancer cells with variable initial expression of cytosolic Bcl-2. **c** The effect of Bax expression on apoptosis and MOMP. **d** The role of reduced expression of cytochrome c (CYCS) on apoptosis. **e** The effect of Smac expression on apoptosis

The expression of XIAP is a critical factor in a cancer cell undergoing type I versus II apoptosis and was altered to determine its effects on Yoda1-mediated TRAIL sensitization (Fig. 6a)^32^. Over-expressed XIAP abolished both cPARP generation and MOMP (Fig. 6a). As XIAP expression is lowered, MOMP that occurs at a faster rate in correlation with cPARP generation. At the normal expression level of XIAP, 1*10^5^ molecules per cell, the cancer cell is reliant on MOMP for apoptosis. When XIAP is under-expressed, MOMP occurs faster, but apoptosis is reached prior to MOMP (Fig. 6a).

Cytosolic Bcl-2 expression had a major effect on MOMP and apoptosis (Fig. 6b). When Bcl-2 was over-expressed, increased calcium could not enhance apoptosis or MOMP as calpain activation was unable to sufficiently cleave Bcl-2 (Fig. 6b). Reducing Bcl-2 expression enhanced the rate of MOMP and cPARP generation. As Bcl-2 expression was lowered the cell was still reliant on MOMP for apoptosis, since MOMP preceded amplified cPARP generation (Fig. 6b). This was further demonstrated by reducing Bax expression to 0 molecules per cell at which point cPARP amplification could not occur even with Bcl-2 deleted. Bcl-2 under-expression removed the need for calpain activation, as this did not affect cPARP generation (Supplementary Fig. 8).

Over-expression of Bax enhanced cPARP generation and reduced the time until MOMP occurred (Fig. 6c). Over-expression of Bax still required calpain to induce enhanced cPARP generation (Supplementary Fig. 9). When Bax was under-expressed, the calcium-TRAIL-induced apoptosis was completely ablated along with MOMP.

Cytochrome c and Smac expression was also altered in the simulation. As cytochrome c and Smac expression was reduced, the level of cPARP generated was also reduced (Fig. 6d, e). Smac under-expression had a much more significant effect on cPARP generation, where a decline from 1 × 10^5^ to 1 × 10^4^ molecules per cell switched off cellular apoptosis in 24 h (Fig. 6e). For cytochrome c, a reduction of 3 orders of magnitude was needed for a similar effect on cPARP generation (Fig. 6d).

### Computational model: cancer cell heterogeneity and calcium-TRAIL response

**Fig. 7:**
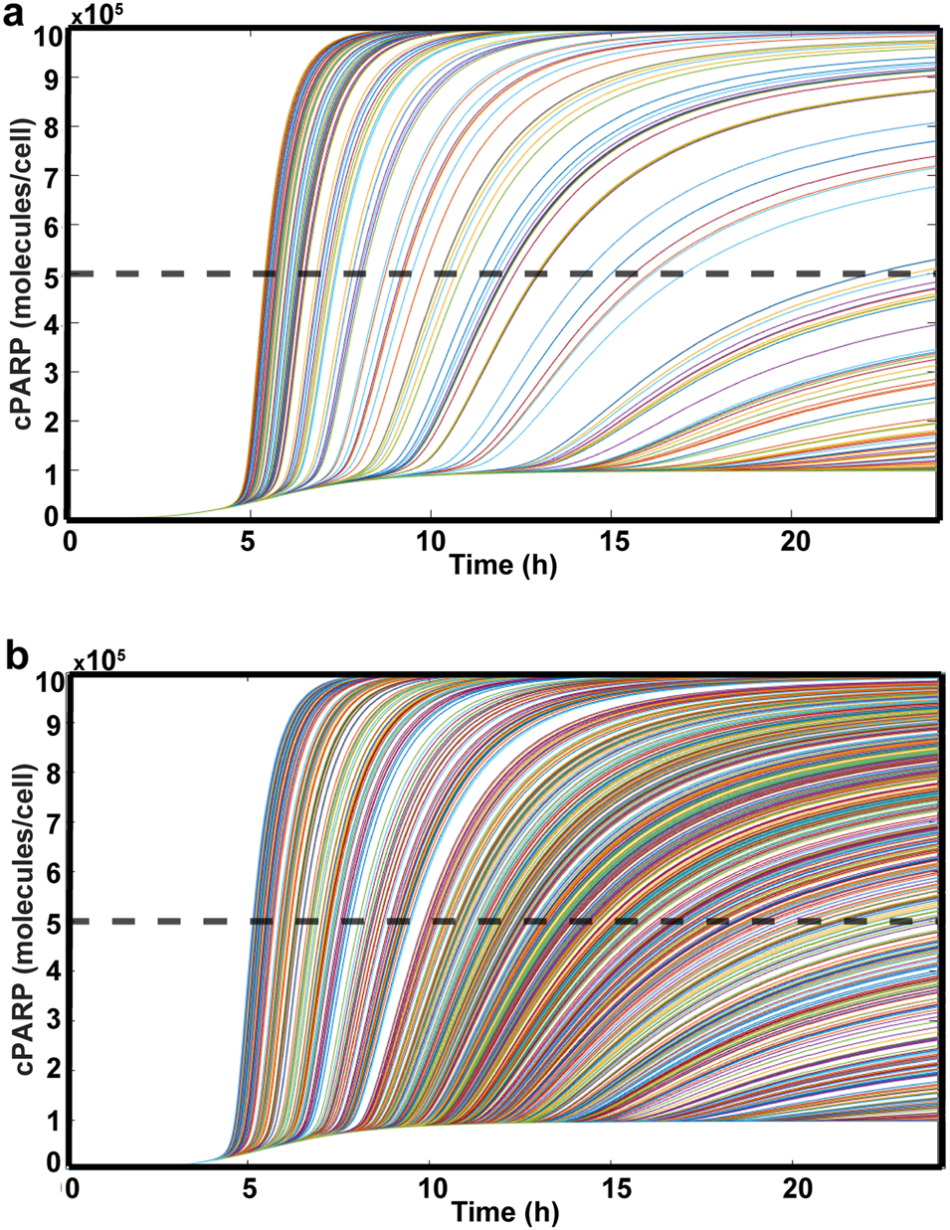
Apoptosis of randomly generated cell populations. **a** Apoptosis simulation of cancer cells with normal-random cytosolic Bcl-2 expression (mean: 1.09 × 10^8^, SD: 1.04 × 10^9^, median: 1.04 × 10^6^) and treated with TRAIL and no increased calcium. Estimated cell viability was 72%. **b** Simulation of random population of cancer cells when treated with TRAIL and increased calcium. Estimated cell viability was 31%. Each line represents an individual cell

The computational model predicts that for the initial conditions specified, cancer cells treated with only TRAIL will not undergo apoptosis, but will when treated with increased calcium and TRAIL. Experimentally, cell death did occur for cancer cells when treated with only TRAIL (Fig. 2b). Altering the initial conditions of various proteins in this simulation changes the need for increased calcium and calpains in TRAIL-mediated apoptosis (Fig. 6). Cells within a tumor and cancer line populations are heterogeneous, partially explaining why an entire cell population is not killed by Yoda1-TRAIL treatment^33,34^. To account for this heterogeneity, a random population of cells was generated by creating a random-normal distribution of cytosolic Bcl-2, as this altered the necessity of calcium (Fig. 6b). The simulated cancer cell population was treated with TRAIL only or with TRAIL and increased calcium for 24 h (Fig. 7). The simulation predicted a cell viability of 72% for cells treated with TRAIL only and 31% for cells treated with increased calcium and TRAIL (Fig. 7). The predicted cell viabilities were greater than what was measured experimentally, but the TRAIL sensitization predicted by the simulation was 56.9%, coinciding with the experimental sensitization of 59.2% at 24 h (Fig. 2).

The elements of the computational model that lend themselves to direct comparison with the experiments generally showed good consistency. For example, both experiments and theory indicated MOMP to be a necessary component of TRAIL sensitization by Yoda1. The results also agree that mitochondrial dysfunction is reliant on the presence of both TRAIL and Yoda1 (Figs. 3 and 4). Bax under-expression in the simulation was found to remove the sensitizing effects of increased calcium (Fig. 6c). This is consistent with the lack of TRAIL sensitization found in DU145 cells (Fig. 2e). Finally, through the generation of a random population of cells, similar levels of TRAIL sensitization were observed in silico compared to the sensitization in experiments (Fig. 7).

The computational model also led to other mechanistic insights not tested experimentally. The simulation showed that cytosolic Bcl-2, XIAP, caspase 3 and other protein expression regulated when a cancer cell would become sensitized to TRAIL via Yoda1 and the degree of sensitization (Figs. 5b and 6a, b). For example, Smac, XIAP, and cytosolic Bcl-2 all heavily regulated sensitization, whereas cytochrome c required a significant reduction in expression to achieve a similar effect (Figs. 5 and 6).

## Discussion

Previously, our lab successfully used TRAIL to treat circulating tumor cells in mice by taking advantage of the shear stress present in the circulatory environment^35,36^. However, there is currently no method of translating this shear stress-sensitizing pathway to primary tumors^37^. Our results indicate that Piezo1 activation played a significant role in the shear stress sensitization of PC3 cells to TRAIL-mediated apoptosis (Fig. 1). Piezo1 activation was recreated under static conditions using Yoda1. Through Yoda1 shear stress sensitization of cancer cells to TRAIL was translated to static conditions, but Yoda1 also sensitized HUVECs to TRAIL-mediated apoptosis (Fig. 2, Supplementary Fig. 5). To utilize Yoda1 and TRAIL’s clinical potential, targeted delivery would be required.

The Yoda1-TRAIL treated cells exhibited an increase of ~2-fold in mitochondrial depolarization and MOMP occurrence when compared to DMSO-TRAIL treated cells, revealing the role of mitochondrial dysfunction in TRAIL sensitization (Fig. 3)^38^. This dysfunction is hypothesized to be due to calpain activation, as calpain inhibition reduced Yoda1-mediated TRAIL sensitization of PC3 cells and calpain activation is linked to Bax activation (Fig. 2f)^23,24^. This mechanism is further supported by previous studies that show Piezo1 activation leads to the downregulation of Bcl-2 and upregulation of calpain activity^21,39^.

The MOMP observed in this study was only correlated with Bax activation (Fig. 3f, g). However, DU145 cells had minimal TRAIL sensitization and are null in Bax^40^. DU145 cells have been previously sensitized to TRAIL through mPTP induction^41^. This strongly supports our mechanism that Yoda1 and TRAIL induce MOMP by Bax activation and not mPTP opening. For future work to conclusively show this, Bax expression could be induced in DU145 cells to analyze if it allows for Yoda1-TRAIL sensitization.

The mechanism of how Yoda1 sensitizes cancer cells to TRAIL is potentially more complex than calpains reducing Bcl-2 activity. Minor amounts of TRAIL sensitization were observed for DU145 cells. Calpain-mediated apoptosis is not solely reliant on Bax-induced MOMP. Calpains activate caspase 12, which leads to increased caspase 3 activity, potentially inducing the small sensitization seen in DU145 cells^42^. Also, calpeptin did not completely abolish the Yoda1-TRAIL cytotoxicity in PC3 cells (Fig. 2f). Calcium influx could also be sensitizing cancer cells to TRAIL by activating calcineurin, another calcium-activated protein that modulates Bcl-2 activity^43^.

The experimental results of this study informed a computational model we developed from the Albeck-Sorger model^44^. The Albeck-Sorger model is a computational model that simulated apoptosis of cancer cells in response to TRAIL. To account for the sensitization to TRAIL induced by Yoda1, additions to the model were made. The updated computational model includes crosstalk between TRAIL and increased calcium by including caspase 3 degradation of calpastatin and calpain activation by calcium which cleaves Bcl-2 and truncates Bid (Fig. 4). The computational model was used to make multiple predictions, some which agreed with experimental results, and others that are yet to be experimentally confirmed. For instance, XIAP as a determining factor of TRAIL sensitization by Yoda1. Testing these predictions experimentally would be valuable in further validating the mechanism and could lead to new targets for inducing TRAIL sensitization therapeutically. Additionally, it would be insightful to randomize the protein expression of many proteins, not just Bcl-2, as cellular heterogeneity extends to multiple proteins^45^.

The utility of this computational model is not limited to the interactions between TRAIL and Yoda1 on cancer cells. Other activators of calpains such as ibulocydine, a CDK inhibitor, and cisplatin, a common chemotherapy, have been previously used to enhance TRAIL-mediated apoptosis^46,47^. The current computational model could be slightly altered to model these combinations as well.

In this study, we have successfully determined the mechanism of shear stress sensitization of cancer cells to TRAIL-mediated apoptosis using shear stress or Yoda1 and TRAIL. A computational model was developed to further explore the sensitization mechanism. Lastly, Yoda1 successfully translated the shear stress sensitization mechanism to static conditions in PC3, MDA-MB-231, and COLO 205 cells (Fig. 2e).

## Materials and methods

### Cell culture

Colorectal adenocarcinoma cell line COLO 205 (ATCC #CCL-222), prostate adenocarcinoma cell lines PC3 (ATCC #CRL-1435) and DU145 (ATCC #HTB-81), and breast adenocarcinoma cell line MDA-MB-231 (ATCC #HTB-26), were purchased from American Type Culture Collection (Manassas, VA, USA). COLO 205 and PC3 cells were cultured in RPMI 1640 cell culture medium (Invitrogen, Grand Island, NY, USA), DU145 cells were cultured in EMEM cell culture medium (Invitrogen), and MDA-MB-231 cells were cultured in DMEM cell culture medium (Invitrogen). Media was supplemented with 10% (v/v) fetal bovine serum and 1% (v/v) PenStrep, all purchased from Invitrogen. HUVECs were purchased from Lonza (Basel, Switzerland) and used from passages 4–5. HUVECs were cultured in Medium 199 (Invitrogen) and supplemented with EGM endothelial cell growth medium SingleQuots kit (Lonza). COLO 205, PC3, DU145, MDA-MB-231, and HUVECs cells were incubated under humidified conditions at 37 °C and 5% CO_2_, and were not allowed to exceed 90% confluence.

### Preparation of cells for fluid shear stress studies

PC3 cells were washed in Ca^2+^ and Mg^2+^ free DPBS (Invitrogen, Carlsbad, CA, USA) and then treated with Accutase (Sigma Aldrich, St. Louis, MO, USA) for 5–10 min at 37 °C before handling. PC3 cells were washed with Ca^2+^ and Mg^2+^ free DPBS at 300 × *g* for 5 min. Cells were resuspended in media at a concentration of 0.5 × 10^6^ cells/mL prior to performing fluid shear stress studies.

For TRAIL studies, cells were treated with 0.250 µg/mL recombinant human TRAIL (Peprotech, Rocky Hill, NJ, USA) and 10 µM GsMTx-4 (Alomone Labs, Jerusalem, Isreal) prior to the application of fluid shear stress.

### Cone-and-plate viscometer assay

To study the fluid shear stress response of PC3 cells in a controlled, uniform environment, studies were conducted using a cone-and-plate device consisting of a stationary plate underneath a rotating cone maintained at room temperature (RT) as described previously^16^. The design of the cone-and plate-viscometer allows a uniform shear rate to be applied to the cell suspension volume. PC3 cells were treated with 2.0 dyn/cm^2^, 10 µM GsMTx-4, and 250 ng/mL TRAIL for 4 h. TRAIL sensitization due to shear stress was calculated under GsMTx-4 treatment and GsMTx-4 treatment conditions using the following equations:

$$\begin{array}{l}\mathrm {TRAIL}\,\mathrm {Sensitization}\left( \% \right) = \\ \frac{{\left( {\% \mathrm {Cells},\mathrm {Shear}\,\mathrm {stress}} \right)\, - \,\left( {\% \mathrm{Cells,Shear}\,\mathrm {stress,TRAIL}} \right)}}{{\% \mathrm{Cells,Shear}\,\mathrm {stress}}}\end{array}$$

or for GsMTx-4 treatment

$$\begin{array}{l}{\mathrm {TRAIL}\,\mathrm {Sensitization}}\left( \% \right) = \\ \frac{{\left( {\% {\mathrm {Cells,Shear}\,\mathrm {stress,GsMTx4}}} \right)\, - \,\left( {\% {\mathrm {Cells,Shear}\,\mathrm {stress,TRAIL,GsMTx4}}} \right)}}{{\left( {\% {\mathrm {Cells,Shear}\,\mathrm {stress,GsMTx4}}} \right)}}\end{array}$$

After treatment, cells were washed thoroughly in Ca^2+^ and Mg^2+^ free DPBS and analyzed for cell death using an Annexin-V assay.

Cancer cells with Piezo1 knockdown were also assessed for shear stress-induced TRAIL sensitization.

### TRAIL-and-Yoda1 cytotoxicity assay

COLO 205, PC3, DU145, MDA-MB-231, and HUVECs were seeded on 12 well plates and incubated overnight at 37 °C to allow cells to adhere to the plate surface. Cells were then treated with 1–50 µM Yoda1 (Tocris, Minneapolis, MN, USA) or 0.01–0.5% DMSO (vehicle control), and TRAIL (COLO 205: 10 ng/mL, PC3: 50 ng/mL, DU145: 100 ng/mL, MDA-MB-231: 50 ng/mL, HUVEC: 100 ng/mL) for 24 h to identify an EC curve for Yoda1. PC3 cells were treated with 10 µM Yoda1 or 0.1% DMSO, and 50 ng/mL TRAIL for 1–24 h. PC3 cells were also transfected with siRNA against Smac, cytochrome c, and a nontargeting negative control. After transfection, the cells were treated with 10 µM Yoda1 or 0.1% DMSO, and 50 ng/mL TRAIL. Cancer cell TRAIL sensitization responses were determined by comparing samples exposed to DMSO and Yoda1 conditions using the following equation:

$$\begin{array}{l}{\mathrm {TRAIL}\,\mathrm {Sensitization}}\left(\% \right) = \\ \frac{{\left( {\% {\mathrm {Cells,TRAIL}\,\& \,\mathrm {DMSO}}} \right)\, -\, \left( {\% {\mathrm {Cells,TRAIL}\,\& \,\mathrm {Yoda1}}} \right)}}{{\left( {\% {\mathrm {Cells,TRAIL}\,\& \,\mathrm {DMSO}}} \right)}} \ast 100\% \end{array}$$

The sensitization equation applies to all cancer cell lines labeled for apoptosis and necrosis for all magnitudes of Yoda1 and DMSO, durations, and transfections.

PC3 cells were treated with Piezo1 siRNA (Thermo Fisher Scientific, Waltham, MA, USA) in combination with Yoda1 and TRAIL to inhibit Piezo1 activation in response to Yoda1.

PC3 cell death was inhibited using 50 µM bongkrekic acid (BKA) (MilliporeSigma, Burlington, MA, USA), 1 µM cyclosporin a (CsA) (MilliporeSigma), 1 µM calpeptin (Tocris), and 5 µM Bax channel blocker (BCB) (Tocris) in addition to Yoda1 and TRAIL.

After treatment, supernatants of the cell cultures were collected. Adherent cells were washed with Ca^2+^ and Mg^2+^ free DPBS and lifted with Accutase. The lifted cells were then added to cell culture supernatants. The samples were analyzed for cell death using an Annexin-V assay.

### Annexin-V apoptosis assay

FITC-conjugated Annexin-V (BD Pharmingen, San Diego, CA, USA) and propidium iodide (PI) (BD Pharmingen) were used to assess cell apoptosis and necrosis. The manufacturer’s instructions were followed to prepare samples for flow cytometric analysis. Viable cells were identified as being negative for both Annexin-V and PI, early apoptotic cells as positive for Annexin-V only, late apoptotic cells were positive for both Annexin-V and PI, and necrotic cells were positive for PI only.

Cells were incubated for 15 min with Annexin-V reagents at RT in the absence of light and immediately analyzed using a Guava easyCyte 12HT benchtop flow cytometer (MilliporeSigma). Flow cytometry plots were analyzed using FlowJo software (FlowJo, Ashland, OR, USA). The following control samples were used to calibrate the instrument: unlabeled cell samples to evaluate the level of autofluorescence and adjust the instrument accordingly, and cell samples labeled individually with Annexin-V and PI to define the boundaries of each cell population.

### JC-1 assay

PC3 cells were seeded onto 12 well plates and incubated overnight at 37 °C to allow cells to adhere. Cells were then treated with 10 µM Yoda1 or 0.1% DMSO, and 50 ng/mL TRAIL. After treatment, the cells were collected and incubated for 20 min at 37 °C with JC-1 dye (Invitrogen) according to the manufacturer’s directions. The cells were then thoroughly washed with Ca^2+^ and Mg^2+^ free DPBS and JC-1 fluorescence was assessed via flow cytometry. Cells with depolarized mitochondria were identified as having low JC-1 red fluorescence and cells with healthy mitochondria were identified as having high red fluorescence.

### Calcium influx

2 × 10^5^ PC3 cells were collected and incubated for 30 min with 1 µM Fluo-4 and 2 µM Fura Red (Invitrogen) at 37 °C. The cells were washed in Ca^2+^ and Mg^2+^ free DPBS and resuspended in HBSS with Ca^2+^ and Mg^2+^ and allowed to incubate for 30 min. PC3 cells were then treated with varying concentrations of Yoda1 and immediately analyzed via flow cytometry to assess real time influx of calcium. Ratiometric fluorescence was calculated by dividing Fluo-4 fluorescence by Fura Red fluorescence. Cells with higher ratiometric fluorescence were identified as having increased intracellular calcium.

### Cell transfection

siRNA oligonucleotides against human Smac (siSmac), Cytochrome C (siCYCS), Piezo1 (siPiezo1), and a nontargeting negative control (Scr) were purchased from Thermo Fisher Scientific. PC3 cells were plated on 12 well plates with mixtures of siRNA, Lipofectamine RNAiMAX reagent (Invitrogen), and Opti-MEM added to each well for a 30 nM siRNA solution. After 48 h of transfection, the culture media was changed to RPMI 1640.

### Western blot

After PC3 cells were transfected with siRNA oligonucleotides, they were lysed with laemmli buffer and subjected to sodium dodecyl sulfate-polyacrylamide gel electrophoresis (SDS-PAGE) [7% (w/v) for Piezo1, 15% (w/v) for Smac and cytochrome c] and transferred to PVDF membrane. After transfer, membranes were blocked with 5% bovine serum albumin (Millipore Sigma) in Tris-buffered saline supplied with 0.1% Tween (Thermo Fisher Scientific). Primary antibodies were prepared at 1:1000 dilution at 5% bovine serum albumin in the case of Piezo1 (Abcam ab128245) and Smac (Cell Signaling 15108) antibody, or at 1:5000 dilution in 5% bovine serum albumin in the case of GAPDH (Millipore MAB374) and cytochrome c (Abcam ab133504). Anti-rabbit or anti-mouse secondary antibodies conjugated to horseradish peroxidase (Rockland, Pottstown, PA, USA) were prepared at 1:2000 dilution in 5% bovine serum albumin. Membranes were imaged with West Pico or Dura (Thermo Fisher Scientific) per their respective protocols, using an ImageQuant LAS-4000 system (GE Healthcare, Chicago, IL, USA).

### Piezo1 expression flow cytometry

COLO 205, PC3, DU145, and MDA-MB-231 cells were stained with a rabbit polyclonal human Piezo1 IgG antibody (Novus Biologicals, Centennial, CO, USA, NBP1-78446) or a rabbit polyclonal Isotype IgG antibody (Santa Cruz Biotechnology, Dallas, TX, USA, SC-66937). Cells were then stained using a secondary antibody goat anti-rabbit IgG Alexa Fluor 633 (Invitrogen A-21070). Piezo1 expression was analyzed using flow cytometry.

### Confocal microscopy

PC3 cells were seeded onto glass coverslips that were previously coated with poly-L-lysine (MilliporeSigma). Cells were allowed to grow for 48 h and then treated with TRAIL at a concentration of 50 ng/mL. Additionally, the test group was treated with 10 µM of Yoda1 and the vehicle control group received DMSO. Cells were treated for 24 h. Cells were fixed for 15 min with 4% paraformaldehyde in PBS (Electron Microscopy Sciences Hatfield, PA, USA), and then permeabilized using 1% Triton X-100 (MilliporeSigma T8787) in PBS. Blocking was done for 2 h with 5% goat serum (Thermo Fisher Scientific) and 5% bovine serum albumin in PBS. Cell staining was performed in the blocking serum. Slides were stained with a Bax antibody (6A7) (Invitrogen MA5-14003). Secondary staining was carried out with an Alexa Fluor 488 goat anti-mouse IgG (H+L) (Thermo Fisher Scientific A11029), in addition to ActinRed 555 ReadyProbes reagent (Invitrogen R37112). All antibodies were diluted in the blocking solution at a ratio of 1:100. Primary staining was done overnight at 4 °C. Secondary staining was performed for 30 min at room temperature. All slides were stained with DAPI (Invitrogen D1306) for 30 min at room temperature in the blocking solution at 1:500. Washes were done twice between each step for 5 min each using 0.02% Tween20 in PBS. Slides were assembled using 10 μL of Vectrashield antifade mounting media (Vector Laboratories, Burlingame, CA, USA). Confocal imaging was performed using an LSM 880 (Carl Zeiss, Oberkochen, Germany) with a 63x/1.40 Plan-Apochromat Oil, WD = 0.19 mm objective (Carl Zeiss). Image analysis was performed using FIJI. Intensity measurements in the green channel (active Bax) were quantified for each cell. This was then divided by the corresponding blue channel (DAPI) intensities for each cell.

### MOMP assay

MOMP was measured using the MitoProbe Transition Pore Assay Kit (Thermo Fisher Scientific) and flow cytometry as the kit directed. 2 µM Calcein-am was added to cells treated with combinations of DMSO, Yoda1, and TRAIL. Next, CoCl_2_ was added to quench cytosolic calcein, or calcein in permeabilized mitochondria. The cells were allowed to incubate for 20 minutes. The cells were then washed once and analyzed via flow cytometry. Reduced calcein fluorescence was considered to be indicative of mitochondrial permeability.

### Statistical analysis

Data sets were plotted and analyzed using Prism 8 (GraphPad software, San Diego, CA, USA). Two-tailed unpaired *t*-test was used for comparisons between two groups with *p* < 0.05 considered significant. One-way ANOVA was used for comparing multiple groups with *p* < 0.05 considered significant. At least three independent replicates were used for each experiment.

### Computational model

Apoptosis modeling was carried out in MATLAB. The systems of ODEs were solved using the ‘ode15s’ function of MATLAB. All model figures were prepared using MATLAB.

### Apoptosis model

The apoptosis model was modified from Albeck-Sorger’s model (**44**) of TRAIL-mediated apoptosis^44^. The Albeck-Sorger model was updated to include calcium, calpain, and calpastatin to model Yoda1’s sensitizing effects. Calcium-activated calpain activity, which was inhibited by calpastatin. Calpastatin was degraded by caspase 3, releasing calpain to degrade Bcl-2 and cleave Bid to its active form. Figure 4a displays a schematic of the model. Supplementary Table 1 displays the biochemical reactions and associated rate constants. Supplementary Table 2 shows the non-zero initial conditions. The model is a series of ODEs that determine when apoptosis takes place using cPARP as an indicator. A cell was considered apoptotic when the cPARP concentration reached 5*10^5^ or half the initial condition of PARP.

For each given reaction, the biochemical equation is represented by one of the following general mass-action paradigms:

1 $$\begin{array}{c}E + S\begin{array}{*{20}{c}} {\mathop { \to }\limits^{k_{ + i}} } \\ {\mathop { \leftarrow }\limits_{k_{ - i}} } \end{array}E:S\mathop { \to }\limits^{K_{ + i}} E + P \\ \left\{ {\begin{array}{*{20}{l}} {\frac{{d\left[ E \right]}}{{dt}} = - k_{ + i}\left[ E \right]\left[ S \right] + k_{ - i}\left[ {E:S} \right] + K_{ + i}[E:S]} \\ {\frac{{d\left[ S \right]}}{{dt}} = - k_{ + i}\left[ E \right]\left[ S \right] + k_{ - i}[E:S]} \\ {\frac{{d\left[ {E:S} \right]}}{{dt}} = k_{ + i}\left[ E \right]\left[ S \right] - k_{ - i}\left[ {E:S} \right] - K_{ + i}[E:S]} \\ {\frac{{d\left[ P \right]}}{{dt}} = K_{ + i}[E:S]} \end{array}} \right.\end{array}$$

2 $$\begin{array}{c}E + S\begin{array}{*{20}{c}} {\mathop { \to }\limits^{k_{ + i}} } \\ {\mathop { \leftarrow }\limits_{k_{ - i}} } \end{array}E:S\mathop { \to }\limits^{K_{ + i}} P \\ \left\{ {\begin{array}{*{20}{l}} {\frac{{d\left[ E \right]}}{{dt}} = - k_{ + i}\left[ E \right]\left[ S \right] + k_{ - i}[E:S]} \\ {\frac{{d\left[ S \right]}}{{dt}} = - k_{ + i}\left[ E \right]\left[ S \right] + k_{ - i}[E:S]} \\ {\frac{{d\left[ {E:S} \right]}}{{dt}} = k_{ + i}\left[ E \right]\left[ S \right] - k_{ - i}[E:S]} \\ {\frac{{d\left[ P \right]}}{{dt}} = K_{ + i}[E:S]} \end{array}} \right.\end{array}$$

3 $$\begin{array}{l}E + S\begin{array}{*{20}{c}} {\mathop { \to }\limits^{k_{ + i}} } \\ {\mathop { \leftarrow }\limits_{k_{ - i}} } \end{array}E:S \\ \left\{ {\begin{array}{*{20}{c}} {\frac{{d\left[ E \right]}}{{dt}} = - k_{ + i}\left[ E \right]\left[ S \right] + k_{ - i}[E:S]} \\ {\frac{{d\left[ S \right]}}{{dt}} = - k_{ + i}\left[ E \right]\left[ S \right] + k_{ - i}[E:S]} \\ {\frac{{d\left[ {E:S} \right]}}{{dt}} = k_{ + i}\left[ E \right]\left[ S \right] - k_{ - i}[E:S]} \end{array}} \right.\end{array}$$

*E* represents an enzyme or other protein that reacts with its substrate or binding partner *S* to form *E:S* or to form product *P*, depending on the reaction. *k*_+*i*_, *k*_−*i*_, and *K*_+*i*_ represent forward, backward, and catalytic rate constants, respectively.

The cytosolic and mitochondrial compartments are assumed to be well mixed. The transport of molecules between the two compartments is represented by the differential equation:

4 $$\frac{{d[x_1]}}{{dt}} = k_{ + i}\left[ {x_1} \right] - k_{ - i}\left[ {x_2} \right]$$

where [*x*] represents the number of molecules in each compartment^44^.

### Random population simulation

To generate a random population of cells treated with TRAIL and increased calcium, the expression of cytosolic Bcl-2 was modeled as a random-normal distribution.

## Supplementary information

Supplementary Table 1

Supplementary Table 2

find_Td.m

TRAIL_init_calcium.m

testPiezo1.m

Duration.m

cellDeathPopulation.m

Supplementary Figure 1

Supplementary Figure 2

Supplementary Figure 3

Supplementary Figure 4

Supplementary Figure 5

Supplementary Figure 6

Supplementary Figure 7

Supplementary Figure 8

Supplementary Figure 9

Author contributions document

## Data Availability

The codes used in this study are provided as supplemental files with this article. The authors request that these programs should not be modified or distributed without attribution to this published work.
