# Supplementary material for: Activation of Piezo1 sensitizes cells to TRAIL-mediated apoptosis through mitochondrial outer membrane permeability
Source: Cell Death Dis. 2019 Nov 4;10(11):837. doi: 10.1038/s41419-019-2063-6 (PMC6828775; doi:10.1038/s41419-019-2063-6)
Supplement: Supplementary file 1 — Supplementary Table 1 [file 41419_2019_2063_MOESM1_ESM.docx]

**Supplementary Table 1:** Reactions and rate constants.

| **Reaction** | $\boldsymbol{k}_{\boldsymbol{i}}$ | $\boldsymbol{k}_{\boldsymbol{-i}}$ | $\boldsymbol{k}_{\boldsymbol{c}}$ | **Ref.** |
| --- | --- | --- | --- | --- |
| $L+R\leftrightarrow L:R \to R^{*}$ | $4*{10}^{-6}$ | $5*{10}^{-3}$ | $1*{10}^{-5}$ | (1) |
| $flip+DISC \leftrightarrow flip:Disc$ | $5*{10}^{-7}$ | $5*{10}^{-4}$ | $NA$ | (1) |
| $pC8+DISC \leftrightarrow pC8:DISC \to C8+DISC$ | $5*{10}^{-7}$ | $5*{10}^{-4}$ | $1$ | (1) |
| $C8+BAR \leftrightarrow C8:BAR$ | $5*{10}^{-8}$ | $5*{10}^{-4}$ | $NA$ | (1) |
| $pC3+C8\leftrightarrow pC3:C8 \to C3+C8$ | $5*{10}^{-8}$ | $5*{10}^{-4}$ | $1$ | (1) |
| $pC6+C3\leftrightarrow pC6:C3\to C6+C3$ | $5*{10}^{-7}$ | $5*{10}^{-4}$ | $1$ | (1) |
| $pC8+C6\leftrightarrow pC8:C6\to C8+C6$ | $6*{10}^{-9}$ | $5*{10}^{-4}$ | $1$ | (1) |
| $XIAP+C3\leftrightarrow XIAP:C3\to XIAP+{C3}_{ub}$ | $2*{10}^{-7}$ | $1*{10}^{-4}$ | $1*{10}^{-4}$ | (1) |
| $PARP+C3\leftrightarrow PARP:C3\to cPARP+C3$ | $1*{10}^{-7}$ | $1*{10}^{-3}$ | $1$ | (1) |
| $Bid+C8\leftrightarrow Bid:C8\to tBid+C8$ | $5*{10}^{-7}$ | $1*{10}^{-4}$ | $1$ | (1) |
| $tBid+Bcl2c\leftrightarrow tBid:Bcl2c$ | $1*{10}^{-8}$ | $1*{10}^{-4}$ | $NA$ | (1) |
| $Bax+tBid\leftrightarrow Bax:tBid\to aBax+tBid$ | $1*{10}^{-9}$ | $1*{10}^{-4}$ | $1$ | (1) |
| $aBax\leftrightarrow MBax$ | $0.01$ | $0.01$ | $NA$ | (1) |
| $MBax+Bcl2\leftrightarrow MBax:Bcl2$ | $1*{10}^{-7}$ | $1*{10}^{-4}$ | $NA$ | (1) |
| $MBax+MBax\leftrightarrow Bax2$ | $1*{10}^{-7}$ | $1*{10}^{-4}$ | $NA$ | (1) |
| $Bax2+Bax2\leftrightarrow Bax4$ | $1*{10}^{-7}$ | $1*{10}^{-4}$ | $NA$ | (1) |
| $Bax4+Bcl2\leftrightarrow Bax4:Bcl2$ | $1*{10}^{-7}$ | $1*{10}^{-4}$ | $NA$ | (1) |
| $Bax4+Mito\leftrightarrow Bax4:Mito\to AMito$ | $1*{10}^{-7}$ | $1*{10}^{-4}$ | $1$ | (1) |
| $AMito+mCytoc\leftrightarrow AMito:mCytoc\to AMito+ACytoc$ | $2*{10}^{-7}$ | $1*{10}^{-4}$ | $10$ | (1) |
| $AMito+mSmac\leftrightarrow AMito:mSmac\to AMito+ASmac$ | $2*{10}^{-7}$ | $1*{10}^{-4}$ | $10$ | (1) |
| $ACytoc\leftrightarrow cCytoc$ | $0.01$ | $0.01$ | $NA$ | (1) |
| $APAF+cCytoc\leftrightarrow APAF:cCytoc\to APAF^{*}$ | $5*{10}^{-8}$ | $1*{10}^{-4}$ | $1$ | (1) |
| $APAF^{*}+pC9\leftrightarrow Apop$ | $5*{10}^{-9}$ | $1*{10}^{-4}$ | $NA$ | (1) |
| $Apop+pC3\leftrightarrow Apop:pC3\to Apop+C3$ | $5*{10}^{-8}$ | $1*{10}^{-4}$ | $1$ | (1) |
| $ASmac\leftrightarrow cSmac$ | $0.01$ | $0.01$ | $NA$ | (1) |
| $Apop+XIAP\leftrightarrow Apop:XIAP$ | $2*{10}^{-7}$ | $1*{10}^{-4}$ | $NA$ | (1) |
| $cSmac+XIAP\leftrightarrow cSmac:XIAP$ | $7*{10}^{-7}$ | $1*{10}^{-4}$ | $NA$ | (1) |
| $calcium+calpain\leftrightarrow calpain:ca$ | $5*{10}^{-10}$ | $1*{10}^{-4}$ | $NA$ | (2) |
| $calcium+calpastatin\leftrightarrow calpastatin^{*}$ | $5*{10}^{-10}$ | $1*{10}^{-4}$ | $NA$ | NA |
| $calpastatin^{*}+calpain:ca\leftrightarrow calpastatin^{*}\to calpain_{bl}$ | $3*{10}^{-7}$ | $1*{10}^{-4}$ | $1$ | NA |
| $C3+calpain_{bl}\leftrightarrow C3:calpain_{bl}\to C3+calpain^{*}$ | $1*{10}^{-7}$ | $1*{10}^{-4}$ | $1$ | NA |
| $calpain^{*}+Bid\leftrightarrow calpain^{*}:Bid\to tBid+calpain^{*}$ | $5*{10}^{-7}$ | $1*{10}^{-4}$ | $1$ | NA |
| $calpain^{*}+Bcl2c\leftrightarrow calpain^{*}:Bcl2c\to calpain^{*}+cBcl2c$ | $2*{10}^{-7}$ | $1*{10}^{-4}$ | $0.1$ | NA |

1. Albeck, J. G., Burke, J. M., Spencer, S. L., Lauffenburger, D. A. & Sorger, P. K. Modeling a Snap-Action, Variable-Delay Switch Controlling Extrinsic Cell Death. *PLOS Biology* **6**, e299 (2008).
2. Hong, J.-Y. *et al.* Computational modeling of apoptotic signaling pathways induced by cisplatin. *BMC Systems Biology* **6**, 122 (2012).
