# Supplementary material for: Activation of Piezo1 sensitizes cells to TRAIL-mediated apoptosis through mitochondrial outer membrane permeability
Source: Cell Death Dis. 2019 Nov 4;10(11):837. doi: 10.1038/s41419-019-2063-6 (PMC6828775; doi:10.1038/s41419-019-2063-6)
Supplement: Supplementary file 2 — Supplementary Table 2 [file 41419_2019_2063_MOESM2_ESM.docx]

**Supplementary Table 2:** Non-zero initial conditions

| **Molecule** | **Basal initial condition (#/CC)** | **Ref.** |
| --- | --- | --- |
| $\left[ \boldsymbol{Ligand} \right]$ | $\boldsymbol{3000=50}\frac{\boldsymbol{ng}}{\boldsymbol{mL}}$ | (1) |
| $\left[ \boldsymbol{Receptor} \right]$ | $\boldsymbol{200}$ | (1) |
| $\left[ \boldsymbol{FLIP} \right]$ | $\boldsymbol{1*1}\boldsymbol{0}^{\boldsymbol{2}}$ | (1) |
| $\left[ \boldsymbol{pC}\boldsymbol{8} \right]$ | $\boldsymbol{2*1}\boldsymbol{0}^{\boldsymbol{4}}$ | (1) |
| $\left[ \boldsymbol{BAR} \right]$ | $\boldsymbol{1*1}\boldsymbol{0}^{\boldsymbol{3}}$ | (1) |
| $\left[ \boldsymbol{pC}\boldsymbol{3} \right]$ | $\boldsymbol{1*1}\boldsymbol{0}^{\boldsymbol{4}}$ | (1) |
| $\left[ \boldsymbol{pC}\boldsymbol{6} \right]$ | $\boldsymbol{1*1}\boldsymbol{0}^{\boldsymbol{4}}$ | (1) |
| $\left[ \boldsymbol{PARP} \right]$ | $\boldsymbol{1*1}\boldsymbol{0}^{\boldsymbol{6}}$ | (1) |
| $\left[ \boldsymbol{Bid} \right]$ | $\boldsymbol{4*1}\boldsymbol{0}^{\boldsymbol{4}}$ | (1) |
| $\left[ \boldsymbol{Bax} \right]$ | $\boldsymbol{1*1}\boldsymbol{0}^{\boldsymbol{5}}$ | (1) |
| $\left[ \boldsymbol{Mito} \right]$ | $\boldsymbol{5*1}\boldsymbol{0}^{\boldsymbol{5}}$ | (1) |
| $\left[ \boldsymbol{Cytochrome C} \right]$ | $\boldsymbol{5*1}\boldsymbol{0}^{\boldsymbol{5}}$ | (1) |
| $\left[ \boldsymbol{Smac} \right]$ | $\boldsymbol{1*1}\boldsymbol{0}^{\boldsymbol{5}}$ | (1) |
| $\left[ \boldsymbol{pC}\boldsymbol{9} \right]$ | $\boldsymbol{1*1}\boldsymbol{0}^{\boldsymbol{5}}$ | (1) |
| $\left[ \boldsymbol{APAF} \right]$ | $\boldsymbol{1*1}\boldsymbol{0}^{\boldsymbol{5}}$ | (1) |
| $\left[ \boldsymbol{Calpain} \right]$ | $\boldsymbol{1*1}\boldsymbol{0}^{\boldsymbol{5}}$ | (2) |
| $\left[ \boldsymbol{Calpastatin} \right]$ | $\boldsymbol{1*1}\boldsymbol{0}^{\boldsymbol{5}}$ | (NA) |
| $\left[ \boldsymbol{cytosolic Bcl}\boldsymbol{2} \right]$ | $\boldsymbol{2*1}\boldsymbol{0}^{\boldsymbol{6}}$ | (1) |
| $\left[ \boldsymbol{mitochondrial Bcl}\boldsymbol{2} \right]$ | $\boldsymbol{2*1}\boldsymbol{0}^{\boldsymbol{4}}$ | (1) |
| $\left[ \boldsymbol{XIAP} \right]$ | $\boldsymbol{1*1}\boldsymbol{0}^{\boldsymbol{5}}$ | (1) |
| $\left[ \boldsymbol{Calcium} \right]$ | $\boldsymbol{1 (\mu M)}$ | (2) |

Non-listed species have initial conditions equal to 0.

1. Albeck, J. G., Burke, J. M., Spencer, S. L., Lauffenburger, D. A. & Sorger, P. K. Modeling a Snap-Action, Variable-Delay Switch Controlling Extrinsic Cell Death. *PLOS Biology* **6**, e299 (2008).
2. Hong, J.-Y. *et al.* Computational modeling of apoptotic signaling pathways induced by cisplatin. *BMC Systems Biology* **6**, 122 (2012).
