# Supplementary figures and images for: Activation of Piezo1 sensitizes cells to TRAIL-mediated apoptosis through mitochondrial outer membrane permeability
Source: Cell Death Dis. 2019 Nov 4;10(11):837. doi: 10.1038/s41419-019-2063-6 (PMC6828775; doi:10.1038/s41419-019-2063-6)

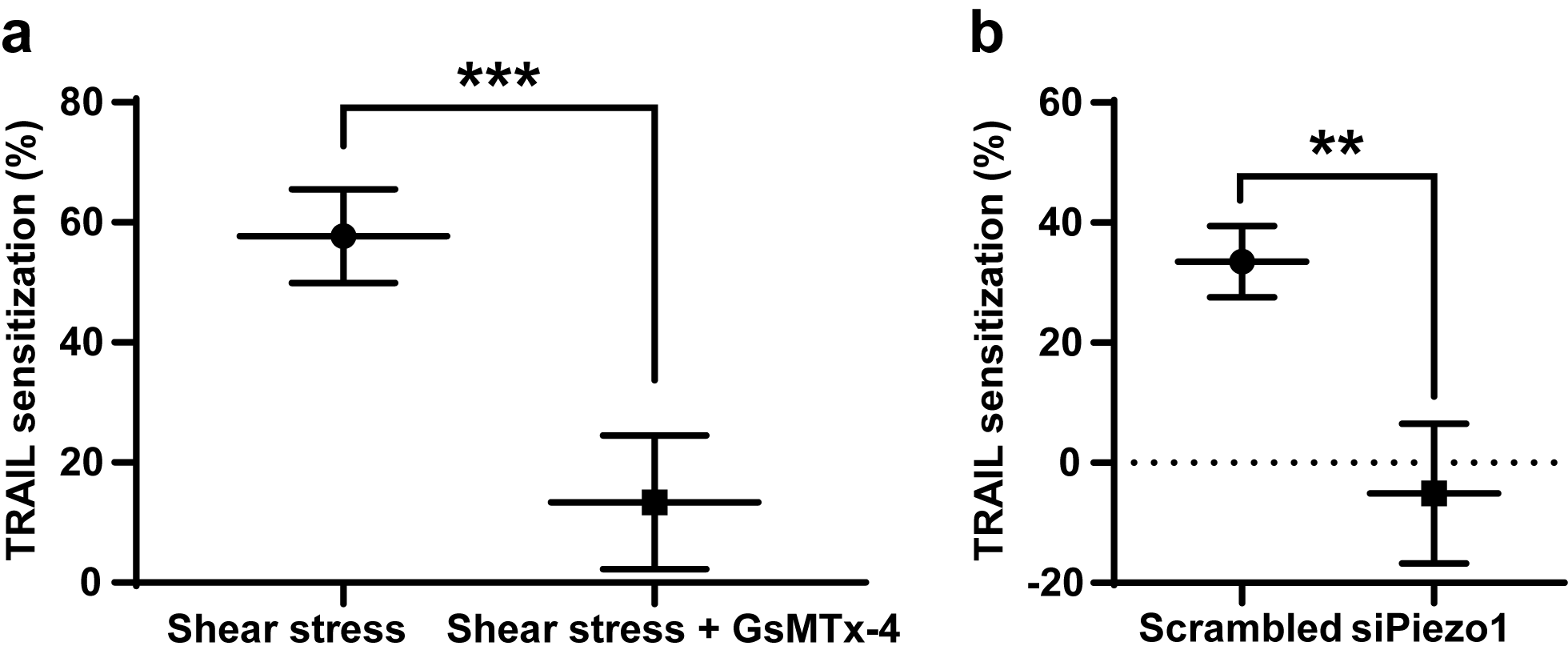

Supplement: Supplementary file 8 — Supplementary Figure 1 [file 41419_2019_2063_MOESM8_ESM.tif]

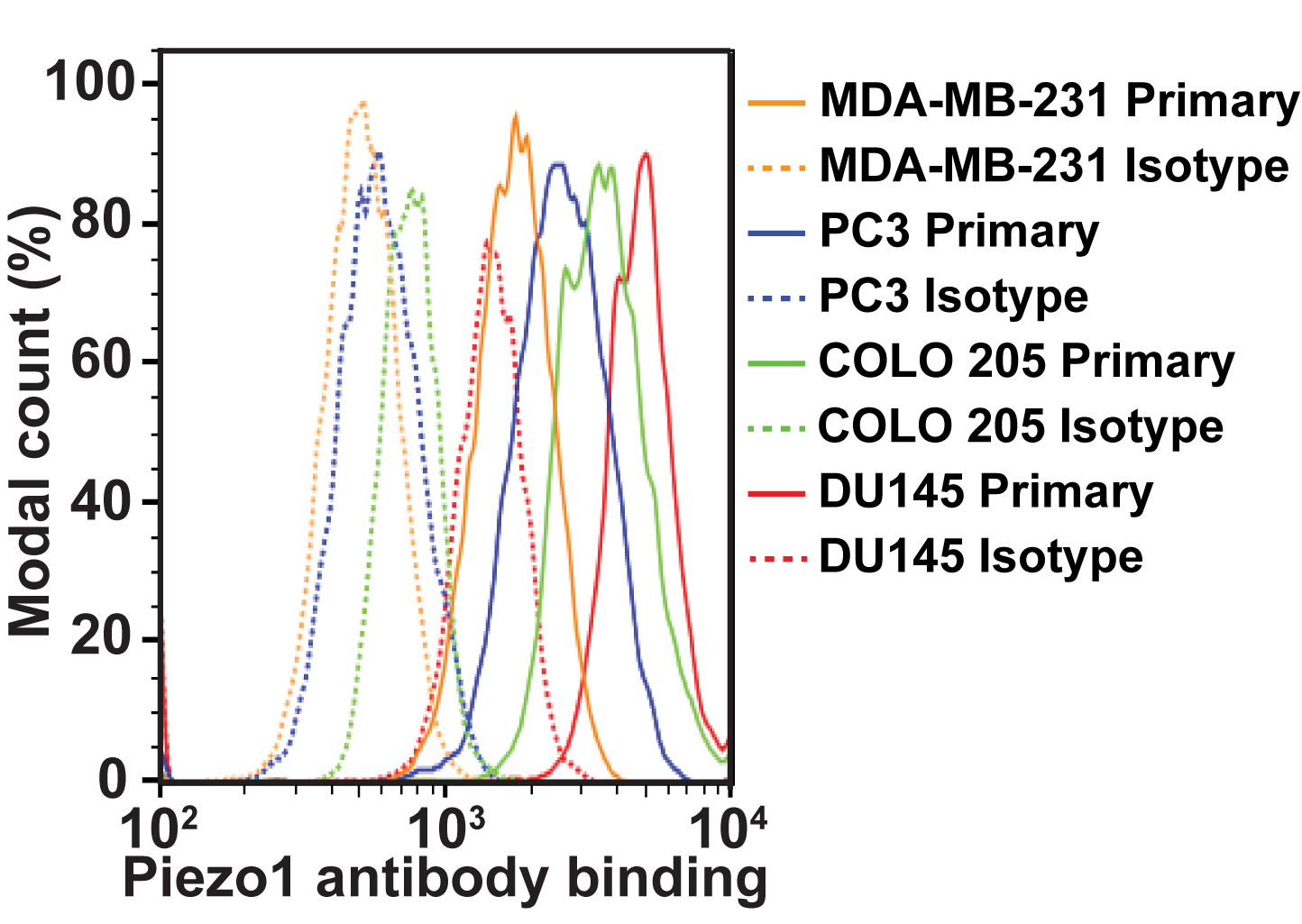

Supplement: Supplementary file 9 — Supplementary Figure 2 [file 41419_2019_2063_MOESM9_ESM.tif]

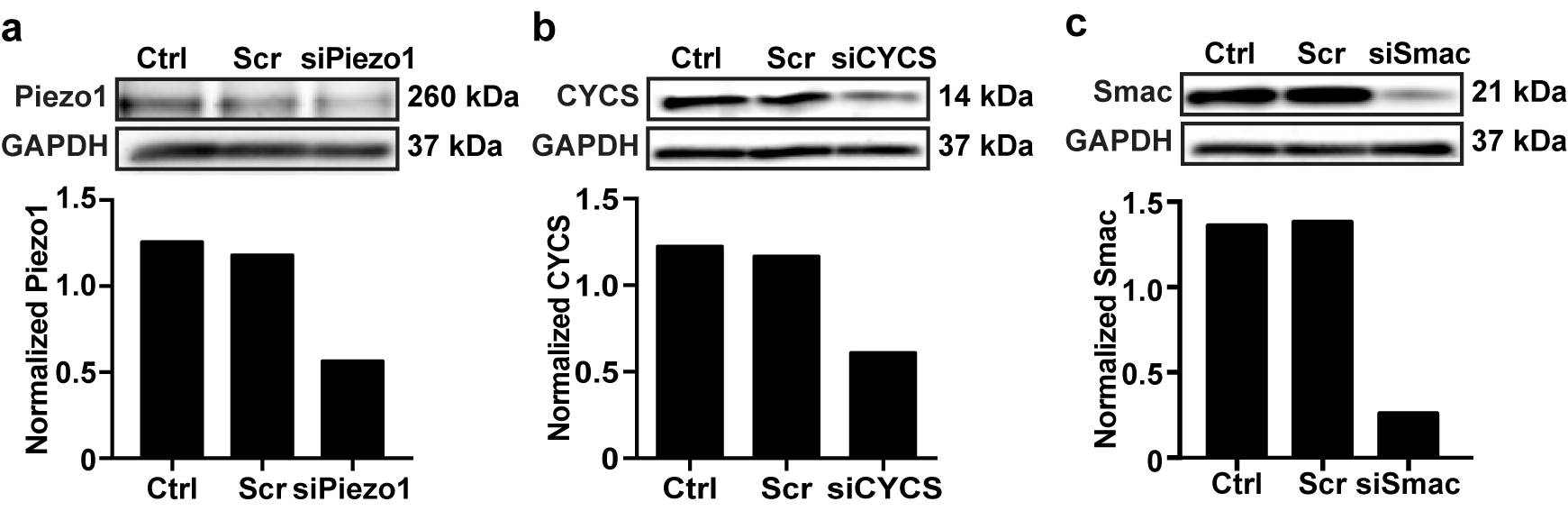

Supplement: Supplementary file 10 — Supplementary Figure 3 [file 41419_2019_2063_MOESM10_ESM.tif]

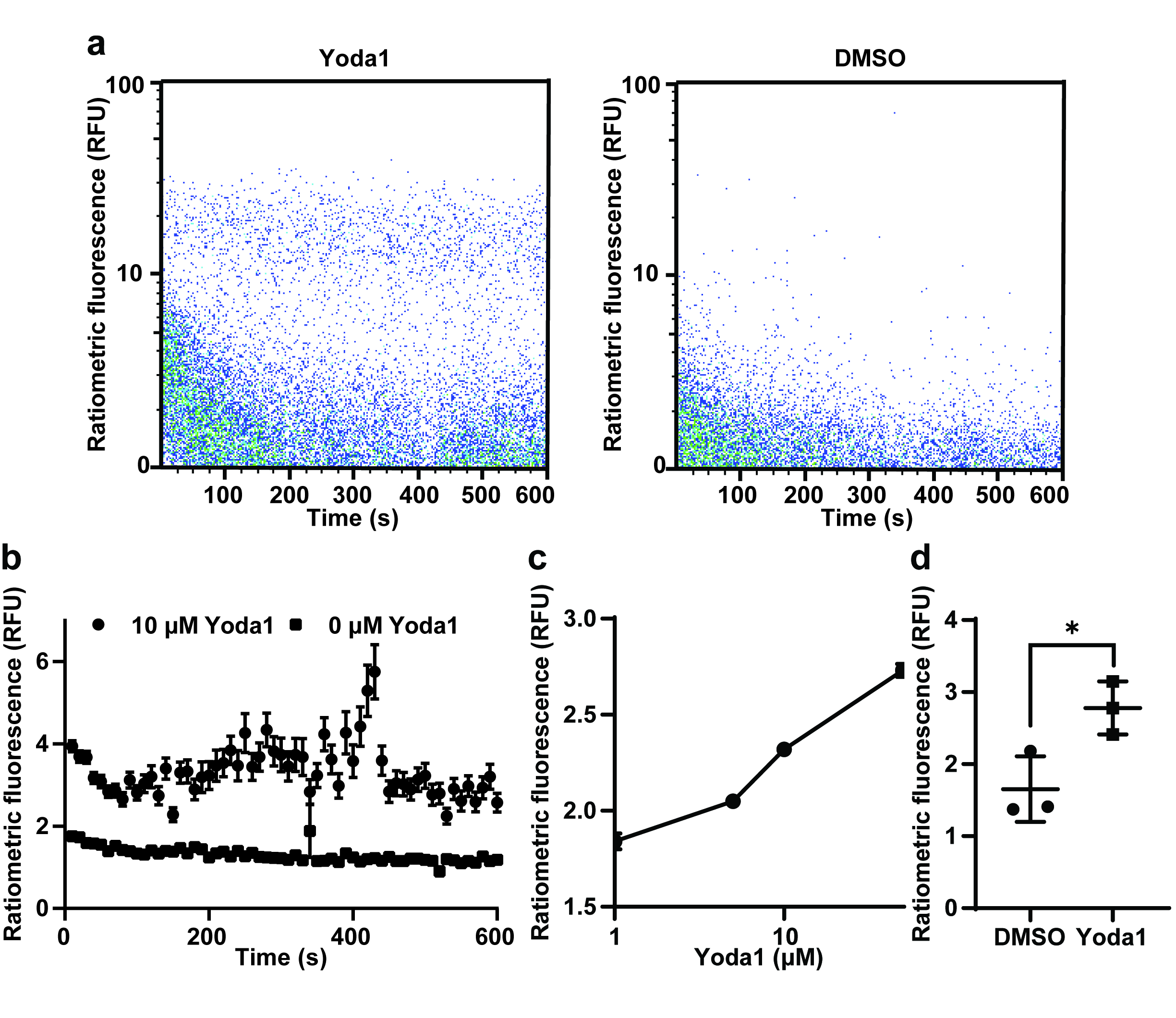

Supplement: Supplementary file 11 — Supplementary Figure 4 [file 41419_2019_2063_MOESM11_ESM.tif]

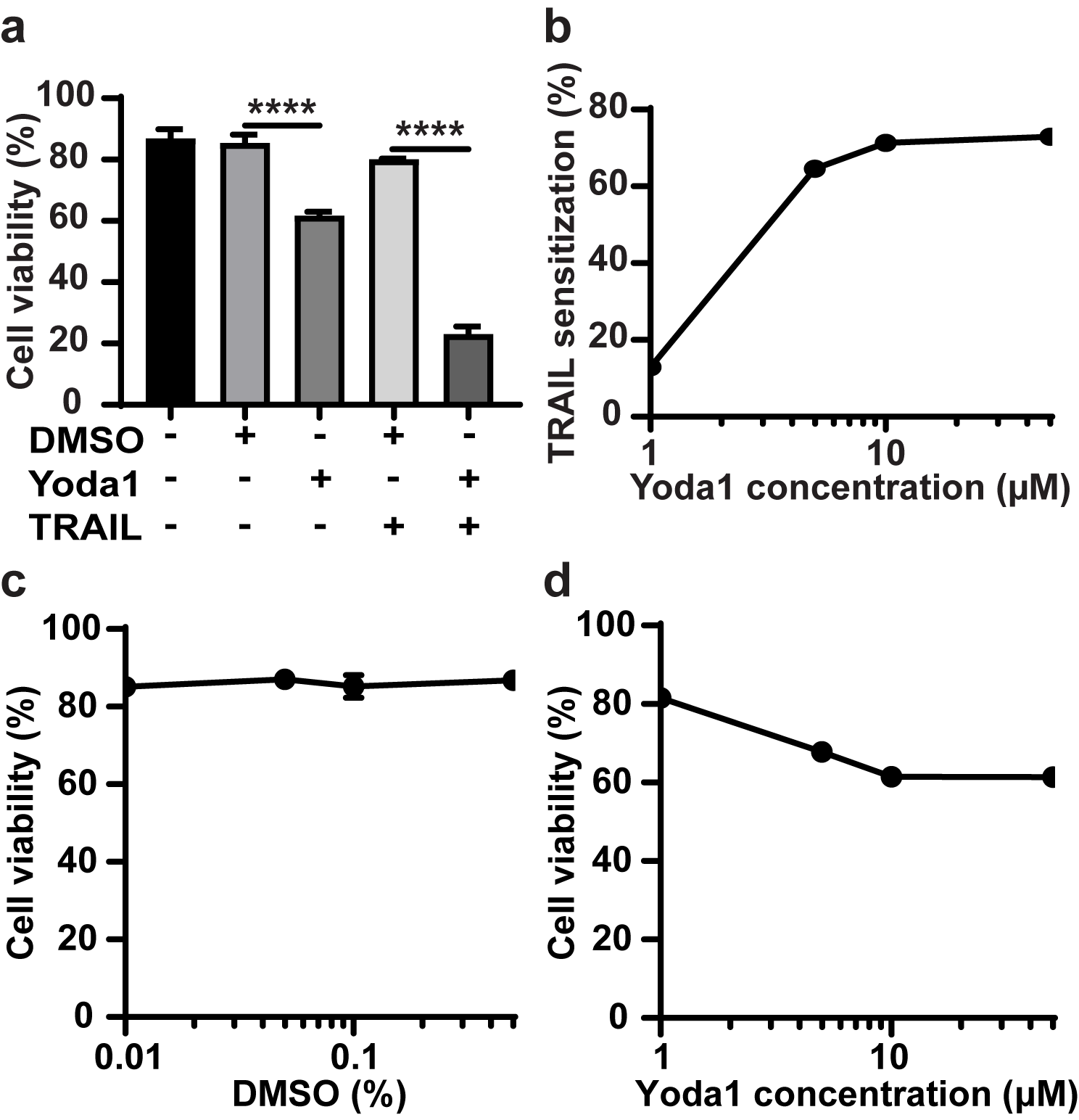

Supplement: Supplementary file 12 — Supplementary Figure 5 [file 41419_2019_2063_MOESM12_ESM.tif]

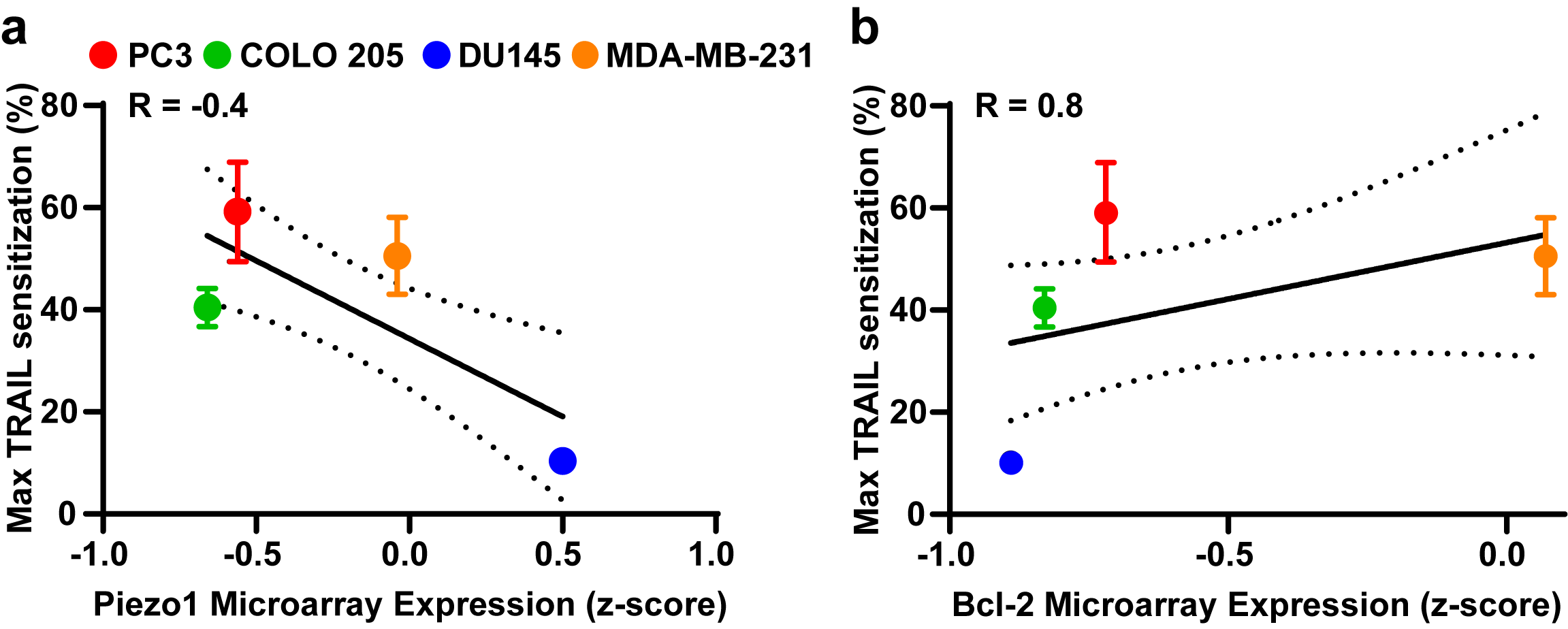

Supplement: Supplementary file 13 — Supplementary Figure 6 [file 41419_2019_2063_MOESM13_ESM.tif]

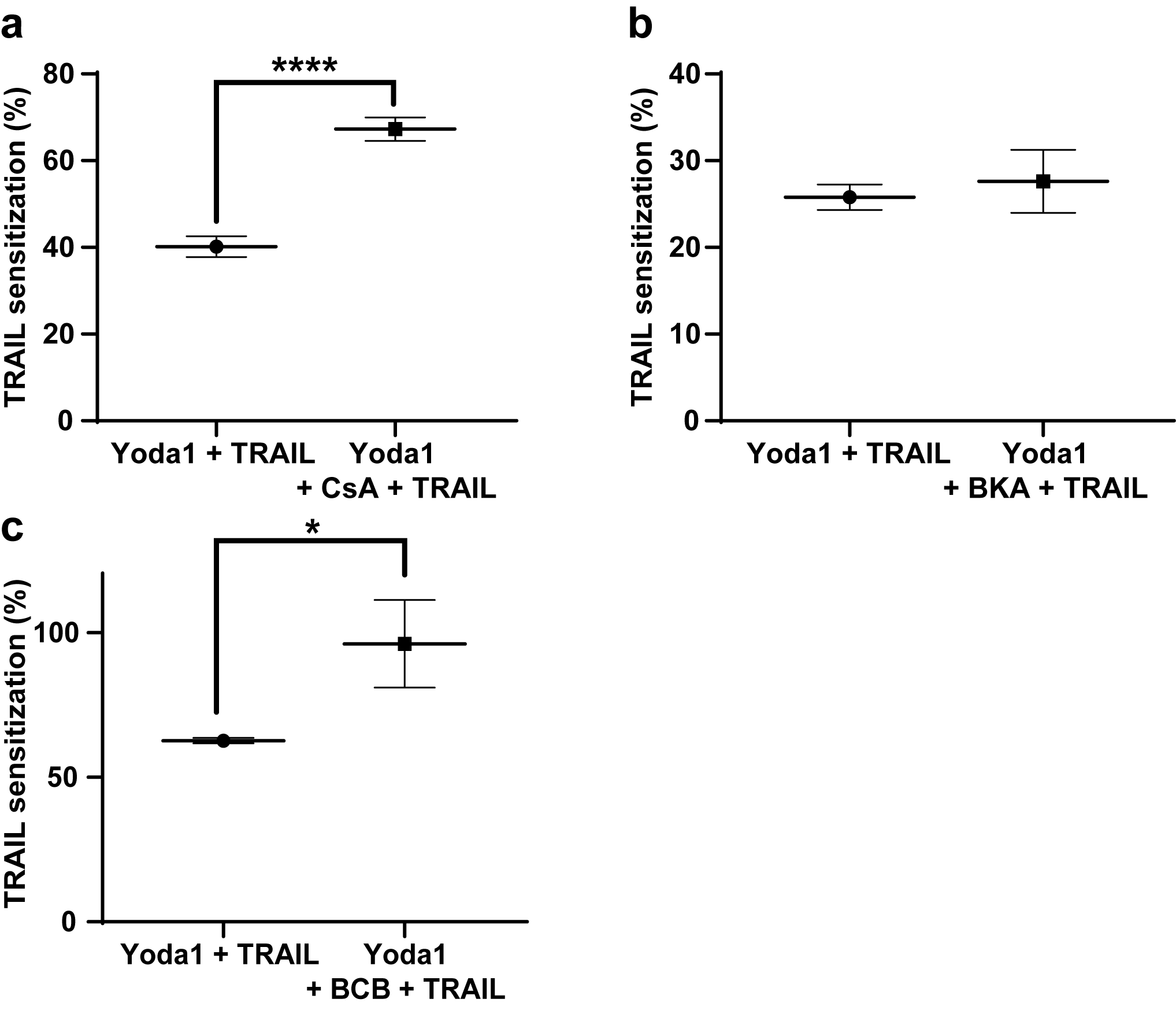

Supplement: Supplementary file 14 — Supplementary Figure 7 [file 41419_2019_2063_MOESM14_ESM.tif]

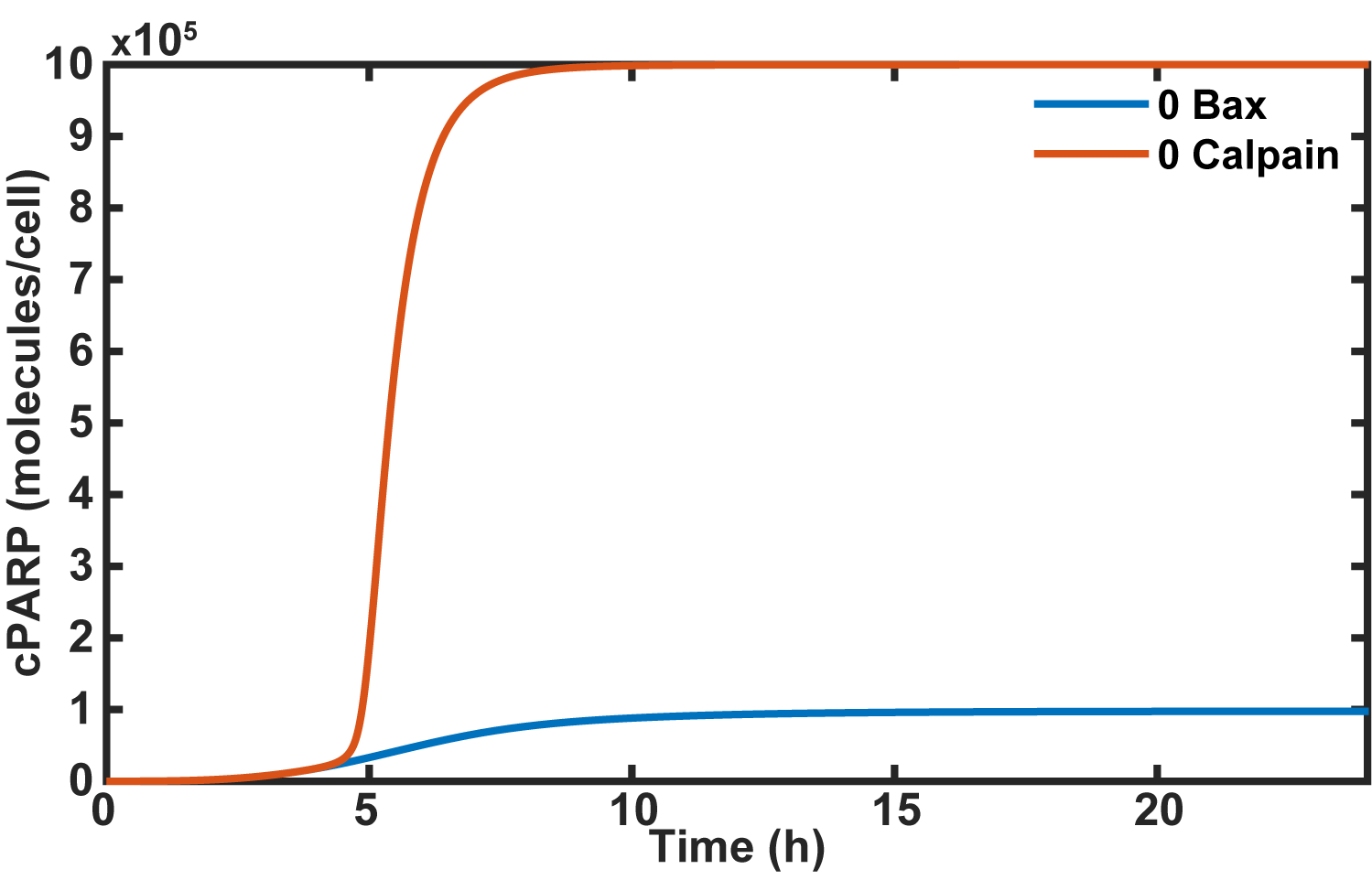

Supplement: Supplementary file 15 — Supplementary Figure 8 [file 41419_2019_2063_MOESM15_ESM.tif]

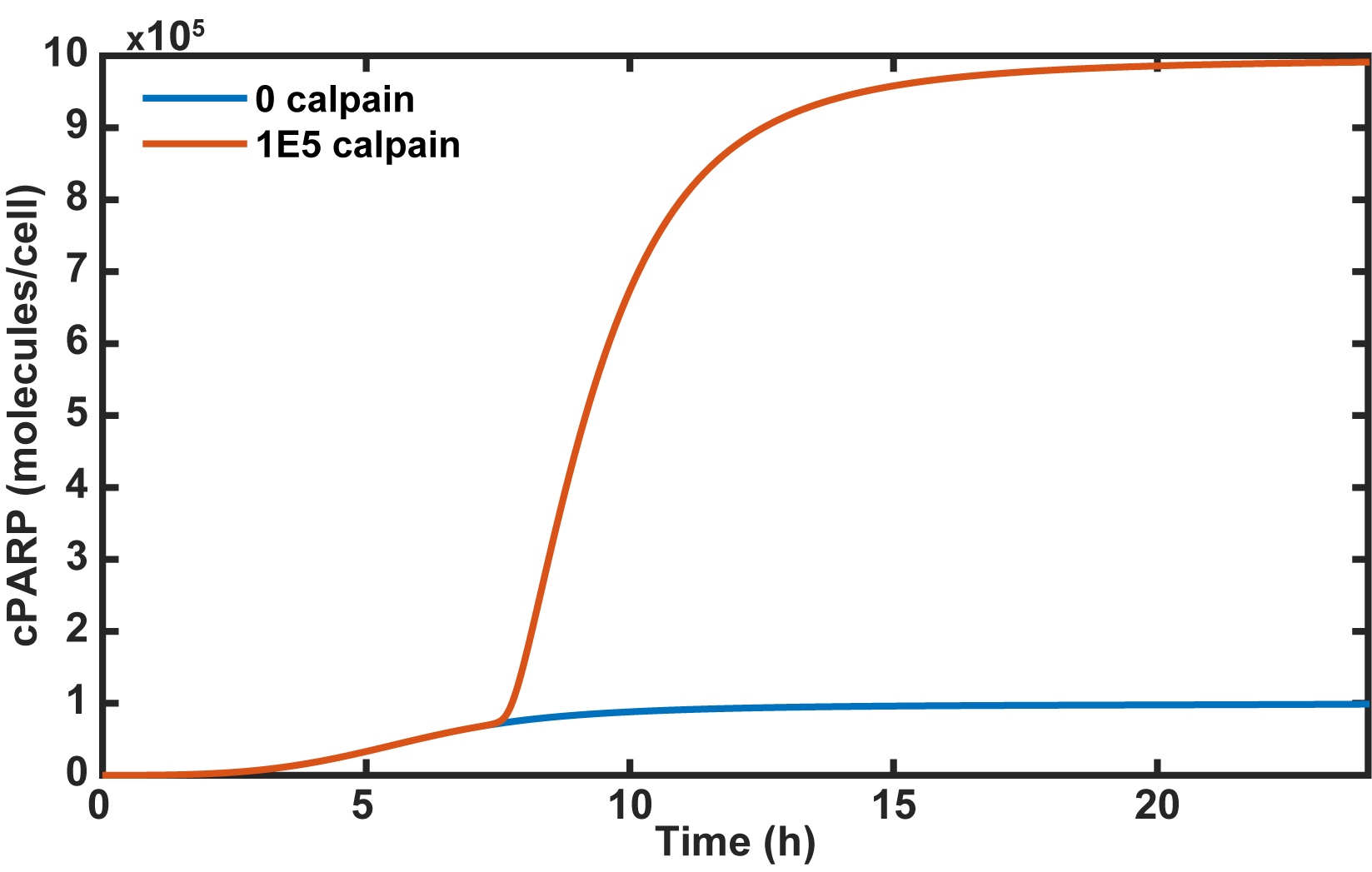

Supplement: Supplementary file 16 — Supplementary Figure 9 [file 41419_2019_2063_MOESM16_ESM.tif]
