# Supplementary material for: Activation of Piezo1 sensitizes cells to TRAIL-mediated apoptosis through mitochondrial outer membrane permeability
Source: Cell Death Dis. 2019 Nov 4;10(11):837. doi: 10.1038/s41419-019-2063-6 (PMC6828775; doi:10.1038/s41419-019-2063-6)
Supplement: Supplementary file 17 — Author contributions document [file 41419_2019_2063_MOESM17_ESM.docx]

**Supplementary Figure 1:** TRAIL sensitization of cancer cells treated with shear stress. **(a)** Shear stress induced TRAIL sensitization of PC3 cells treated with or without 10 µM GsMTx-4 (n=4). **(b)** Shear stress sensitization of PC3 cells to TRAIL when Piezo1 expression was knocked down (n=4). Means and SD are from four independent experiments. Statistical analysis done using two-tailed unpaired t-test. **p<0.01, ***p<0.005.

**Supplementary Figure 2:** Piezo1 expression of MDA-MB-231, PC3, COLO 205, and DU145 cells. Representative experiment of three independent experiments.

**Supplementary Figure 3:** Protein expression of knocked down proteins. **(a)** Piezo1 expression after knockdown. **(b)** Cytochrome c (CYCS) knockdown expression after knockdown. **(c)** Smac expression after knockdown. Representative experiment of three independent experiments.

**Supplementary Figure 4:** Calcium concentration of PC3 cells after treatment with Yoda1. **(a)** Flow plots of intracellular calcium concentration measured by ratiometric fluorescence for PC3 cells treated with Yoda1 or DMSO. **(b)** Real-time analysis of intracellular calcium concentration of PC3 cells treated with 0 or 10 µM Yoda1 (n>100). **(c)** Ratiometric fluorescence of PC3 cells treated with 1 (n=33486), 5 (n=31129), 10 (n=29197), and 50 µM (n=49638) Yoda1. **(d)** Mean ratiometric fluorescence of PC3 cells treated with DMSO or Yoda1 (n=3). **(a)-(c)** Representative experiments of three independent experiments. Means and SEM displayed. **(d)** Mean and SD of three independent experiments. Statistical analysis done using two-tailed unpaired t test. *p<0.05.

**Supplementary Figure 5:** Apoptosis of HUVECs treated with Yoda1 and TRAIL. **(a)** Cell viability of HUVECs treated with 0.1% DMSO or 10 µM Yoda1 and 100 ng/mL TRAIL (n=3). **(b)** TRAIL sensitization of HUVECs treated with 1, 5, 10, and 50 µM Yoda1 (n=3). **(c)** Cell viability of HUVECs treated with DMSO (n=3). **(d)** Cell viability of HUVECs treated with various concentrations of Yoda1 (n=3). Means and SD of three independent experiments. Statistical analysis done using one-way ANOVA. ****p<0.001.

**Supplementary Figure 6:** Correlation of TRAIL sensitization and microarray expression z-scores. **(a)** Correlation of Piezo1 expression with max TRAIL sensitization of PC3, COLO 205, DU145, and MDA-MB-231 cells. **(b)** Correlation of Bcl-2 expression and max TRAIL sensitization. Microarray expression z-scores taken from CBioportal.

**Supplementary Figure 7:** Inhibition of MOMP on Yoda1-induced TRAIL sensitization. **(a)** TRAIL sensitization of PC3 cells treated with 1 µM cyclosporin a (CsA) (n=3). **(b)** TRAIL sensitization of PC3 cells treated with 50 µM bongkrekic acid (BKA) (n=3). **(c)** TRAIL sensitization of PC3 cells treated with 5 µM Bax channel blocker (BCB). (n=3). Means and SD from three independent experiments. Statistical analysis done using two-tailed unpaired t-test. *p<0.05, ****p<0.001.

**Supplementary Figure 8:** Simulation of apoptosis of cancer cells without Bax or calpain expression treated with TRAIL and increased calcium with no Bcl-2 expression.

**Supplementary Figure 9:** Simulation of cancer cells with over-expressed Bax of $1*{10}^{6}$ molecules per cell treated with TRAIL and increased calcium with or without calpain expression

**Supplementary Table 1:** Reactions and rate constants.

1. Albeck, J. G., Burke, J. M., Spencer, S. L., Lauffenburger, D. A. & Sorger, P. K. Modeling a Snap-Action, Variable-Delay Switch Controlling Extrinsic Cell Death. *PLOS Biology* **6**, e299 (2008).
2. Hong, J.-Y. *et al.* Computational modeling of apoptotic signaling pathways induced by cisplatin. *BMC Systems Biology* **6**, 122 (2012).

**Supplementary Table 2:** Non-zero initial conditions

Non-listed species have initial conditions equal to 0.

1. Albeck, J. G., Burke, J. M., Spencer, S. L., Lauffenburger, D. A. & Sorger, P. K. Modeling a Snap-Action, Variable-Delay Switch Controlling Extrinsic Cell Death. *PLOS Biology* **6**, e299 (2008).
2. Hong, J.-Y. *et al.* Computational modeling of apoptotic signaling pathways induced by cisplatin. *BMC Systems Biology* **6**, 122 (2012).
